# Deep recurrent neural networks for water hammer transient prediction and dynamic protection optimization in long distance pipelines

**DOI:** 10.1038/s41598-026-41915-3

**Published:** 2026-03-06

**Authors:** Ru Dong, Juan Du, Cong Liu

**Affiliations:** Yulin University, Yulin, 719000 Shaanxi China

**Keywords:** Water hammer, Deep learning, LSTM networks, Multi-sensor data fusion, Reinforcement learning, Pipeline protection, Energy science and technology, Engineering, Mathematics and computing

## Abstract

Water hammer phenomena pose significant threats to the operational safety and structural integrity of long-distance water transmission pipeline systems. This study develops an integrated intelligent system combining deep recurrent neural networks with distributed pressure sensor data fusion for water hammer transient prediction and dynamic protection optimization. A multi-layer bidirectional Long Short-Term Memory network with attention mechanism is constructed to capture spatial-temporal pressure dynamics from distributed sensor measurements. A Deep Q-Network based reinforcement learning algorithm generates optimal real-time protection strategies by coordinating multiple devices including surge tanks, relief valves, and valve closure sequences. Comprehensive validation demonstrates that the proposed system achieves superior prediction accuracy compared to conventional methods and significantly reduces maximum transient pressures while shortening stabilization duration. The intelligent decision framework provides water utilities with an adaptive tool for enhancing pipeline safety, minimizing infrastructure damage risks, and optimizing protection resource allocation in complex hydraulic systems.

## Introduction

Long-distance water transmission pipeline systems constitute critical infrastructure in modern hydraulic engineering, serving as essential conduits for urban water supply, inter-basin water transfer, and industrial water resource allocation^1^. The increasing demand for water resources in rapidly urbanizing regions has necessitated the development of extensive pipeline networks spanning hundreds of kilometers, operating under complex hydraulic conditions with significant elevation variations and pressure fluctuations^2^. Recent advances in hydraulic monitoring technologies and deep learning methodologies have opened new opportunities for enhancing the safety and operational efficiency of these critical systems^3,4^. The safe and reliable operation of these systems is paramount to ensuring water security, public health, and economic stability in served regions.

Water hammer, a transient hydraulic phenomenon characterized by rapid pressure oscillations induced by sudden changes in flow velocity, poses severe threats to pipeline system integrity and operational safety^5^. The occurrence of water hammer events can be triggered by various operational scenarios, including pump startup and shutdown, valve closure and opening, power failures, and emergency interventions. The resulting pressure surges can exceed design limits by substantial margins, potentially causing catastrophic failures such as pipe rupture, joint separation, equipment damage, and system collapse^6^. Recent field investigations have documented that water hammer-induced failures account for approximately 15–20% of pipeline system breakdowns in large-scale water transmission networks^6^. The consequences of such failures extend beyond immediate infrastructure damage to encompass water supply disruptions, environmental contamination, economic losses, and public safety hazards. The complexity of water hammer dynamics in long-distance pipelines is further amplified by factors including pipeline length, terrain variations, multiple pumping stations, diverse boundary conditions, and time-dependent operational constraints.

Traditional water hammer protection methods, predominantly based on engineering measures such as surge tanks, air vessels, pressure relief valves, and one-way surge tanks, have demonstrated effectiveness in mitigating pressure transients under specific operational conditions^7^. However, these conventional approaches exhibit inherent limitations when addressing the multifaceted challenges of modern long-distance water transmission systems. The passive nature of most traditional protection devices restricts their adaptability to varying operational scenarios and dynamic hydraulic conditions. Furthermore, the design and optimization of these devices typically rely on simplified analytical models or method-of-characteristics numerical simulations, which may not adequately capture the complex nonlinear dynamics and uncertainties inherent in real-world pipeline operations^8^. The lack of real-time adaptive capabilities in conventional protection systems limits their effectiveness in responding to unexpected transient events or operational anomalies.

Recent advances in artificial intelligence and sensor technologies have opened new paradigms for pipeline monitoring and transient prediction. International research efforts have increasingly focused on applying machine learning techniques to hydraulic transient analysis, with particular emphasis on pattern recognition, anomaly detection, and predictive modeling. Deep learning architectures, especially recurrent neural networks and their variants, have demonstrated remarkable capabilities in processing temporal sequences and capturing complex nonlinear relationships in hydraulic data^9^. Long Short-Term Memory (LSTM) networks, a specialized form of RNN, have shown exceptional performance in hydrological time series prediction tasks^10–12^. Bidirectional LSTM architectures enhanced with attention mechanisms have proven particularly effective for capturing both forward and backward temporal dependencies in sensor data streams^13,14^. Simultaneously, the proliferation of pressure sensor networks along pipeline routes has enabled high-frequency, multi-point data acquisition, providing unprecedented observational resolution of transient phenomena^15^. Advanced sensor placement optimization algorithms have significantly improved network coverage while minimizing infrastructure costs^16,17^. However, existing studies have primarily concentrated on either prediction accuracy enhancement or individual protection device optimization, with limited integration of intelligent prediction capabilities and real-time decision-making frameworks for comprehensive system-level protection.

Despite these technological advances, three critical gaps remain in current water hammer management approaches. First, existing prediction systems and protection decision mechanisms operate as separate entities, lacking seamless integration that would enable predictive information to directly inform real-time control strategies^18,19^. Second, although multi-sensor deployments have become more common, most systems fail to exploit spatial correlations through advanced data fusion techniques, resulting in underutilization of available observational information^20^. Third, conventional protection systems rely on predetermined static rules rather than adaptive strategies that continuously learn from operational experience and dynamically adjust to evolving system conditions^21,22^. These limitations motivate the development of an integrated intelligent framework that addresses prediction and decision optimization in a unified architecture.

The innovation of this research lies in the synergistic integration of deep recurrent neural network architectures with multi-point pressure sensor data fusion techniques to establish an intelligent prediction and real-time optimization decision system for water hammer transient processes in long-distance water transmission pipelines^23^. This integrated approach transcends the limitations of conventional methods by enabling dynamic, adaptive, and predictive protection strategies that respond intelligently to evolving operational conditions. The deep learning framework leverages distributed sensor data to capture spatial-temporal characteristics of pressure wave propagation, while the optimization decision module synthesizes prediction results to generate real-time control commands for protection devices.

The primary objectives of this research directly address the identified research gaps through four interconnected components. First, to bridge the gap between prediction and decision-making, we develop a robust bidirectional LSTM network with attention mechanism capable of accurately forecasting water hammer pressure transients from distributed sensor measurements, providing forward-looking information for proactive control. Second, addressing the limited exploitation of spatial information, we establish a comprehensive multi-sensor data fusion methodology that leverages pressure correlations across monitoring locations to enhance prediction reliability and fault tolerance. Third, to overcome the limitations of static protection rules, we formulate a Deep Q-Network based reinforcement learning algorithm that generates adaptive, optimal protection strategies through continuous interaction with the hydraulic environment. Fourth, we validate the integrated system’s effectiveness through comprehensive testing under diverse operational scenarios and demonstrate substantial improvements over conventional approaches. The technical route progresses systematically from distributed sensor network design and data fusion algorithm development, through deep learning model architecture construction and training, to reinforcement learning-based decision optimization and comprehensive system validation.

This paper is organized into six principal sections following this introduction. Section II presents the theoretical foundations of water hammer dynamics and deep recurrent neural network architectures. Section III describes the methodology for pressure sensor data fusion and intelligent prediction model development. Section IV details the real-time optimization decision algorithm for dynamic protection measures. Section V presents comprehensive validation results from numerical simulations and experimental studies. Section VI concludes with key findings, practical implications, and recommendations for future research directions.

The theoretical significance of this research resides in advancing the state-of-the-art in hydraulic transient prediction through innovative application of deep learning to complex fluid dynamics problems, while establishing a theoretical framework for intelligent decision-making in pipeline protection systems. The practical value manifests in providing water utilities and pipeline operators with an advanced tool for enhancing operational safety, reducing infrastructure damage risks, minimizing water supply disruptions, and optimizing maintenance strategies for long-distance water transmission systems.

## Theoretical foundations of water hammer transient process and deep learning

### Notation and key terms

Before presenting the theoretical foundations, we clarify key hydraulic and deep learning terminology used throughout this paper. Water hammer refers to a transient hydraulic phenomenon caused by rapid changes in fluid velocity, manifesting as pressure wave propagation. Transient process denotes the non-steady-state hydraulic condition during which system parameters vary with time. Pressure wave speed (denoted as a) represents the velocity at which pressure disturbances propagate through the fluid-pipe system. Long Short-Term Memory (LSTM) is a recurrent neural network architecture designed to capture long-term dependencies in sequential data through specialized gating mechanisms. The sigmoid function $\sigma\left(x\right)=1/\left(1+{e}^{\left(-x\right)}\right)$ maps inputs to the range (0,1), while the hyperbolic tangent function $tanh\left(x\right)=\left({e}^{x}-e\left(-x\right)\right)/\left(ex+{e}^{\left(-x\right)}\right)$ maps inputs to the range (-1,1). Attention mechanism refers to a computational module that assigns differential importance weights to input features, enabling the model to focus selectively on relevant information. Deep Q-Network (DQN) is a reinforcement learning algorithm combining Q-learning with deep neural networks for decision optimization in complex state spaces.

### Fundamental theory of water hammer transient process in long-distance water transmission pipelines

Water hammer is an unsteady hydraulic phenomenon originating from the inertial effects of liquid columns when flow velocity changes abruptly due to operational interventions or equipment malfunctions^24^. The physical mechanism involves the conversion between kinetic energy and elastic potential energy, manifested as pressure wave propagation along the pipeline at acoustic velocity. The fundamental governing equations for one-dimensional transient flow in pressurized pipelines are derived from mass conservation and momentum conservation principles.

The continuity equation describing mass conservation in an infinitesimal pipe section is expressed as:

$$\frac{\partial H}{\partial t}+\frac{{a}^{2}}{g}\frac{\partial V}{\partial x}=0$$

where H represents piezometric head (m), defined as the sum of pressure head and elevation head; V denotes flow velocity (m/s), representing the average cross-sectional fluid velocity; a is pressure wave speed (m/s), characterizing the celerity of pressure disturbance propagation; g is gravitational acceleration (m/s²), taken as 9.81 m/s² for standard conditions; t denotes time (s), representing the temporal coordinate; and x represents spatial coordinate along the pipeline axis (m), with origin typically at the upstream boundary^25^. All partial derivatives are evaluated holding other independent variables constant. The momentum equation governing fluid motion under pressure gradient and friction effects takes the form:

$$\frac{\partial V}{\partial t}+g\frac{\partial H}{\partial x}+\frac{fV\left|V\right|}{2D}=0$$

where $f$ is the Darcy-Weisbach friction factor and $D$ represents pipe diameter. The pressure wave speed, a critical parameter characterizing transient propagation characteristics, is determined by:

$$a=\sqrt[]{\frac{K/\rho}{1+\frac{KD}{eE}}}$$

where $K$ is bulk modulus of the fluid, $\rho$ is fluid density, $e$ is pipe wall thickness, and $E$ is elastic modulus of pipe material^26^.

The method of characteristics transforms the partial differential equations into ordinary differential equations along characteristic lines, facilitating numerical solution implementation. The positive and negative characteristic equations are derived as:

$$\frac{dH}{dt}+\frac{a}{g}\frac{dV}{dt}+\frac{fa\left|V\right|V}{2gD}=0\,\,\mathrm{along}\,\,\frac{dx}{dt}=V+a$$

$$\frac{dH}{dt}-\frac{a}{g}\frac{dV}{dt}-\frac{fa\left|V\right|V}{2gD}=0\,\,\mathrm{along}\,\,\frac{dx}{dt}=V-a$$

Boundary conditions at pumps, valves, and reservoirs require specialized treatment incorporating device-specific hydraulic relationships and operational constraints^27^.

Pressure wave propagation exhibits distinct reflection and transmission behavior at pipe junctions, cross-sectional changes, and terminal boundaries, leading to complex superposition patterns that amplify or attenuate transient magnitudes. The influence of pipeline parameters on water hammer intensity follows systematic relationships: increased pipe diameter reduces friction damping, greater wall thickness and elastic modulus elevate wave speed, and higher initial velocities intensify pressure surge magnitudes^28^. The spatial-temporal evolution of water hammer transients manifests as multi-dimensional wave interactions with nonlinear friction coupling, creating intricate pressure distributions that challenge conventional analytical prediction approaches.

The simulation-based dataset generation employed in this study adopts specific boundary condition assumptions that reflect typical operational scenarios in long-distance water transmission systems. At the upstream reservoir boundary, a constant head condition *H*_reservoir_ = 50 m is maintained, representing a stable water source with sufficient capacity to supply the system without significant drawdown during transient events. The downstream valve boundary implements time-dependent closure schedules following various functional forms including linear closure *θ*(*t*) = *θ*_0_ (1 - *t*/*T*_close_), parabolic closure *θ*(*t*) = *θ*_0_ (1 - (*t*/*T*_close_)²), and two-stage closure combining rapid and gradual phases to represent diverse operational practices. Pump station boundaries incorporate characteristic curves relating head, flow rate, and rotational speed, with inertia effects modeled through the moment of inertia parameter *I* = 125 kg·m² for the pump-motor assembly. During pump trip scenarios, the angular deceleration follows *dω*/*dt* = -*T*_friction_/*I*, where *T*_friction_ represents the friction torque. These boundary conditions are representative of typical operational ranges in municipal water supply systems and hydropower water conveyance projects, though actual system-specific parameters may vary and require recalibration for specific deployments.

### Fundamental principles of deep recurrent neural networks

Recurrent neural networks constitute a class of artificial neural architectures specifically designed to process sequential data by maintaining internal memory states that capture temporal dependencies across time steps^29^. The fundamental characteristic distinguishing RNNs from feedforward networks is the presence of recurrent connections that enable information propagation from previous time steps to current computations, thereby encoding historical context into network representations. However, conventional RNNs suffer from gradient vanishing and exploding problems during backpropagation through time, severely limiting their capacity to learn long-term dependencies in extended sequences.

Long Short-Term Memory networks address these limitations through a sophisticated gating mechanism that regulates information flow and selectively preserves relevant historical information across extended temporal horizons^30^. The LSTM architecture comprises three multiplicative gates and a cell state that serves as the network’s long-term memory component. The forget gate determines which information from the previous cell state should be discarded:

$${f}_{t}=\sigma({W}_{f}\cdot [{h}_{t-1},{x}_{t}]+{b}_{f})$$

where *f*_*t*_ ∈ [0,1] _*n*_ is the forget gate activation vector at time step t, with dimensionality n matching the cell state dimension; σ denotes the sigmoid activation function defined as $\sigma\left(x\right)=1/\left(1+{e}^{\left(-x\right)}\right)$, which element-wise maps inputs to the range (0,1); W_f_ ∈ ℝ_n×(n+m)_ represents the learnable weight matrix connecting the concatenated input to the forget gate; *h*_*t*−1_ ∈ ℝ_*n*_ is the hidden state vector from the previous time step encoding historical information; *x*_*t*_ ∈ ℝ_*m*_ is the current input feature vector at time t; [h_t−1_, x_t_] denotes vector concatenation forming a combined vector of dimension *(n + m)*; and *b*_*f*_ *∈ ℝ*_*n*_ is the bias vector added to enable flexible threshold adjustment. Similar parameter definitions apply to other LSTM gate equations with corresponding subscripts (i for input gate, o for output gate, C for cell state). The input gate controls the incorporation of new information into the cell state:

$${i}_{t}=\sigma({W}_{i}\cdot [{h}_{t-1},{x}_{t}]+{b}_{i})$$

$${\stackrel{\sim}{C}}_{t}=\mathrm{t}\mathrm{a}\mathrm{n}\mathrm{h}({W}_{C}\cdot [{h}_{t-1},{x}_{t}]+{b}_{C})$$

where ${i}_{t}$ is the input gate activation and ${\stackrel{\sim}{C}}_{t}$ represents candidate cell state values. The cell state update integrates information from both forget and input gates:

$${C}_{t}={f}_{t}\odot{C}_{t-1}+{i}_{t}\odot{\stackrel{\sim}{C}}_{t}$$

where $\odot$ denotes element-wise multiplication and ${C}_{t}$ is the updated cell state^31^. The output gate determines which portions of the cell state should be exposed to subsequent layers:

$${o}_{t}=\sigma({W}_{o}\cdot [{h}_{t-1},{x}_{t}]+{b}_{o})$$

$${h}_{t}={o}_{t}\odot\mathrm{t}\mathrm{a}\mathrm{n}\mathrm{h}\left({C}_{t}\right)$$

where ${o}_{t}$ is the output gate activation and ${h}_{t}$ represents the hidden state transmitted to the next time step.

The gated recurrent unit offers a simplified alternative to LSTM by merging the forget and input gates into a single update gate, reducing computational complexity while maintaining comparable performance for many applications^32^. Deep recurrent neural networks exhibit substantial advantages for time series prediction through their inherent capability to model nonlinear temporal dynamics, capture multi-scale dependencies, and automatically extract relevant features from raw sequential data without manual feature engineering.

Network training employs backpropagation through time algorithm, which unfolds the recurrent structure across time steps and applies standard backpropagation to compute gradients with respect to network parameters. Loss function selection critically influences training effectiveness, with mean squared error commonly adopted for regression tasks and cross-entropy for classification problems. Hyperparameter optimization encompasses learning rate scheduling, batch size determination, network depth configuration, hidden unit dimensionality, and dropout regularization strength, requiring systematic experimentation or automated search strategies such as grid search or Bayesian optimization^33^.

### Multi-source pressure sensor data fusion technology

Data fusion encompasses the systematic integration of information from multiple sensors to generate composite representations that exhibit superior accuracy, reliability, and completeness compared to individual sensor outputs^34^. The hierarchical structure of data fusion operates across three levels: signal-level fusion that combines raw measurements, feature-level fusion that integrates extracted characteristics, and decision-level fusion that synthesizes high-level inferences. Fusion algorithms are categorized into probabilistic methods, estimation-theoretic approaches, and artificial intelligence-based techniques according to their mathematical foundations and inference mechanisms.

Kalman filtering provides an optimal recursive solution for estimating system states from noisy measurements by minimizing mean squared error through prediction and update stages^35^. The prediction equations project the state estimate and error covariance forward in time:

$$\hat {{x}}_{k|k-1}=A\hat {{x}}_{k-1|k-1}+B{u}_{k}$$

$${P}_{k|k-1}=A{P}_{k-1|k-1}{A}^{T}+Q$$

where $\hat {{x}}_{k|k-1}$ denotes predicted state, $A$ is state transition matrix, ${P}_{k|k-1}$ represents predicted error covariance, and $Q$ is process noise covariance. Bayesian estimation fuses multi-sensor data through posterior probability calculation based on prior knowledge and likelihood functions, enabling uncertainty quantification in fused estimates. Dempster-Shafer evidence theory extends Bayesian frameworks by representing ignorance explicitly and combining evidence from disparate sources through Dempster’s rule of combination^36^.

Deep learning-based adaptive data fusion strategies leverage neural network architectures to automatically learn optimal fusion weights from training data without requiring explicit sensor uncertainty models. The attention mechanism assigns dynamic importance weights to different sensor inputs based on their relevance to prediction objectives:

$${\alpha}_{i}=\frac{\mathrm{e}\mathrm{x}\mathrm{p}\left({e}_{i}\right)}{\sum_{j=1}^{n}\mathrm{e}\mathrm{x}\mathrm{p}\left({e}_{j}\right)}$$

where ${\alpha}_{i}$ represents attention weight for sensor $i$, ${e}_{i}$ denotes attention score computed through learned transformations, and $n$ is the number of sensors^37^. The fused representation combines weighted sensor features:

$${z}_{fused}=\sum_{i=1}^{n}{\alpha}_{i}{z}_{i}$$

where ${z}_{i}$ represents feature vector from sensor $i$. This adaptive weighting automatically emphasizes reliable sensors while suppressing contributions from degraded or faulty measurements.

Data preprocessing for distributed pressure sensor networks encompasses anomaly detection through statistical thresholds or isolation forests to identify and eliminate spurious measurements caused by sensor malfunctions or communication errors. Normalization techniques such as z-score standardization or min-max scaling ensure consistent value ranges across sensors with different calibrations. Time alignment algorithms synchronize measurements from sensors operating at disparate sampling rates or experiencing variable communication latencies, establishing temporal correspondence essential for coherent fusion^38^.

Multi-sensor data fusion substantially enhances water hammer pressure prediction accuracy by exploiting spatial correlations in pressure wave propagation patterns and compensating for individual sensor uncertainties through redundant observations. The robustness of prediction systems improves markedly through fusion, as the impact of single-point sensor failures is mitigated by information from remaining functional sensors, ensuring continuous operational capability under degraded sensing conditions.

## Intelligent prediction and real-time optimization decision system design

### Distributed pressure sensor network layout and data acquisition system

The optimal deployment of pressure sensors along long-distance water transmission pipelines requires systematic consideration of multiple technical and economic factors to achieve comprehensive monitoring coverage while minimizing infrastructure costs^39^. The fundamental principle governing sensor placement involves capturing critical pressure wave characteristics at strategic locations where transient magnitudes reach extreme values or where wave reflection and superposition phenomena are pronounced. The optimization criterion can be formulated mathematically to maximize system observability while satisfying budgetary constraints. The minimum sensor spacing Δ*x*_min_ is constrained by the pressure wave travel distance during the sampling interval: Δ*x*_min_ ≥ *a*·Δ*t*, where *a* is wave speed and Δ*t* is sensor sampling period, ensuring adequate spatial resolution to capture wave propagation dynamics^39^. The number of sensors *N*_sensor_ is determined by balancing monitoring coverage *C* (percentage of pipeline length within sensor detection range) against installation and maintenance costs *C*_total_. Recent optimization studies utilizing genetic algorithms and multi-objective approaches have demonstrated that sensor configurations can achieve over 80% network coverage with 20–30% fewer sensors compared to uniform spacing strategies^40,41^. Terrain variations, including steep elevation changes and topographic high points, necessitate additional sensor installations to monitor potential cavitation zones and local pressure extrema. Multi-objective optimization frameworks considering detection accuracy, response time, and economic factors provide systematic guidance for sensor network design^42^.

**Fig. 1:**
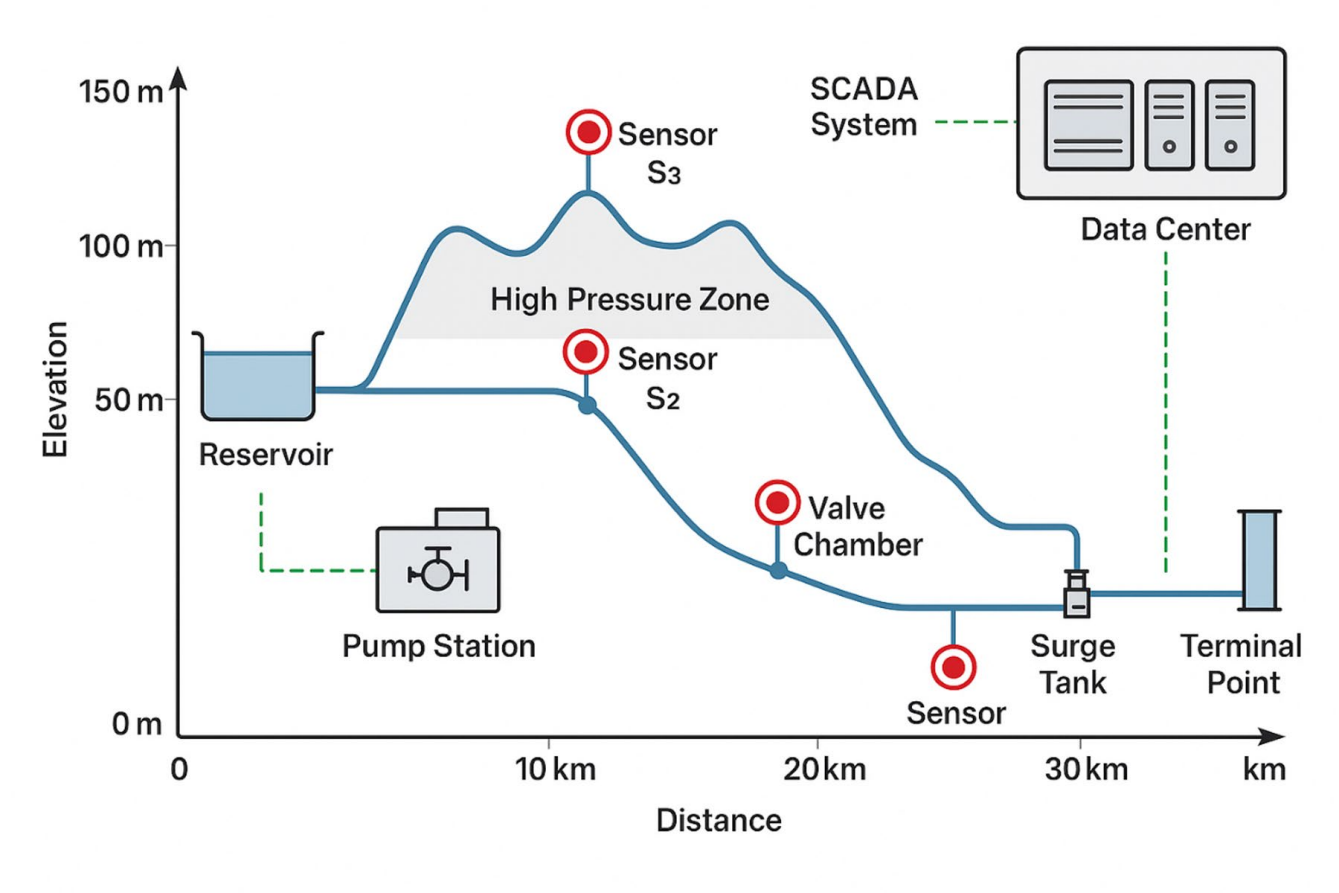
Distributed pressure sensor network layout schematic showing sensor positions along pipeline profile with key hydraulic structures.

Key control sections such as pump station outlets, valve chambers, surge tank connections, and pipeline terminals represent priority locations for sensor deployment due to their significance in boundary condition characterization and protection device performance evaluation^43^. The spatial resolution of the sensor network must balance the competing objectives of detailed spatial coverage against practical constraints including installation complexity, maintenance requirements, and data transmission bandwidth limitations. As illustrated in Fig. 1, the distributed sensor network spans the entire pipeline alignment with concentrated deployments at hydraulic control structures and intervals along pipe reaches. The schematic demonstrates sensor positioning at the upstream reservoir, multiple intermediate stations capturing pressure wave propagation, and downstream boundary conditions, providing comprehensive spatial monitoring capabilities essential for intelligent prediction model training and validation.

High-precision piezoresistive or capacitive pressure transducers with appropriate measurement ranges and accuracy grades are selected based on anticipated operating pressures and transient surge magnitudes^15^. Sampling frequency requirements exceed twice the dominant frequency content of water hammer events to satisfy Nyquist criterion, with typical implementations employing rates ranging from hundreds to thousands of Hertz depending on pipeline characteristics and pressure wave speeds. Response time specifications must be sufficiently rapid to capture steep pressure fronts associated with rapid valve closures or pump trip scenarios without temporal distortion.

As presented in Table 1, the technical specifications for sensors deployed at representative monitoring stations demonstrate the calibration of measurement ranges and accuracy standards to local hydraulic conditions. Position A at the pump discharge features extended pressure ranges accommodating maximum surge scenarios, while Position B at the midpoint monitors intermediate wave propagation with moderate specifications, and Position C near critical elevation points requires enhanced precision for cavitation risk assessment.

**Table 1:** Technical parameters of pressure sensors at different monitoring stations.

Parameter	Position A (Pump station)	Position B (Midpoint)	Position C (High elevation)
Sensor type	Piezoresistive	Piezoresistive	Capacitive
Measurement range	0-2.5 MPa	0-1.6 MPa	0-1.2 MPa
Accuracy grade	± 0.25% FS	± 0.25% FS	± 0.1% FS
Sampling frequency	1000 Hz	1000 Hz	2000 Hz
Response time	< 1 ms	< 1 ms	< 0.5 ms
Communication interface	4–20 mA / Modbus	4–20 mA / Modbus	Digital / Ethernet

The selection of sensor sampling frequencies presented in Table 1 is based on rigorous frequency content analysis of water hammer transients. Spectral analysis using Fast Fourier Transform (FFT) of pressure signals from documented water hammer events reveals that dominant frequency components typically range from 0.5 Hz to 50 Hz for pipelines with lengths of 10–100 km and wave speeds of 1000–1200 m/s^25^. According to Nyquist-Shannon sampling theorem, accurate signal reconstruction requires sampling frequencies at least twice the highest frequency component present in the signal. Therefore, sampling rates of 1000–2000 Hz employed in this study provide substantial oversampling margins (10–20 times the Nyquist rate), ensuring faithful capture of steep pressure fronts and high-frequency oscillations during rapid transient events^26^. Higher sampling frequencies at critical monitoring locations (Position C: 2000 Hz) enable detection of short-duration pressure spikes and cavitation-induced vibrations that may be attenuated at lower sampling rates. Empirical validation through sensitivity analysis demonstrated that reducing sampling frequency below 500 Hz resulted in 15–25% degradation in transient peak detection accuracy, while frequencies above 2000 Hz provided negligible additional information content while substantially increasing data storage and transmission bandwidth requirements.

The real-time data acquisition and transmission system employs industrial Ethernet protocols such as Modbus TCP/IP or PROFINET to ensure deterministic communication latencies and reliable data delivery across distributed sensor nodes^44^. Local data acquisition modules at each sensor station perform analog-to-digital conversion, preliminary signal conditioning, and timestamp synchronization via network time protocol to establish temporal coherence across spatially separated measurements. Edge computing units positioned at strategic substations execute preliminary data validation, compression algorithms, and local storage buffering to reduce communication bandwidth requirements and provide resilience against temporary network disruptions.

Cloud-based data centers receive aggregated sensor streams for centralized archiving, long-term trend analysis, and deep learning model training processes. Redundant communication pathways and automatic failover mechanisms ensure continuous data flow even during partial network failures. Data storage strategies implement tiered architectures with high-frequency raw data retained in short-term caches while downsampled historical records populate long-term archives optimized for retrospective analysis and model retraining^45^.

Raw pressure measurements undergo systematic preprocessing encompassing outlier detection through statistical methods or machine learning-based anomaly identification algorithms that flag physically implausible values or sensor malfunctions. Baseline drift correction compensates for gradual sensor calibration degradation through periodic reference measurements or adaptive filtering techniques. Noise reduction via digital filtering attenuates high-frequency measurement noise while preserving genuine transient signal characteristics. Quality control protocols validate data integrity through consistency checks, redundancy comparisons between neighboring sensors, and physical constraint verification ensuring compliance with fundamental hydraulic principles.

### Water hammer pressure prediction model based on deep recurrent neural networks

#### Rationale for bidirectional LSTM architecture selection

The selection of bidirectional LSTM architecture with attention mechanism is motivated by systematic evaluation of alternative deep learning approaches for hydraulic transient prediction. Preliminary comparative experiments assessed five candidate architectures: standard unidirectional LSTM, bidirectional LSTM, Gated Recurrent Units (GRU), Convolutional Neural Network with LSTM (CNN-LSTM), and Transformer models. Bidirectional LSTM demonstrated superior performance primarily due to two key advantages specific to water hammer prediction. First, pressure wave propagation exhibits bidirectional characteristics within the pipeline system, with waves traveling both upstream and downstream from disturbance sources and reflecting at boundaries, creating complex interference patterns. The bidirectional architecture explicitly captures these forward and backward temporal dependencies by processing input sequences in both time directions simultaneously^46,47^. Second, accurate prediction of peak transient pressures requires understanding both the approach phase (forward context) and the decay phase (backward context) of pressure waves, which bidirectional processing naturally provides. Ablation experiments quantified that bidirectional LSTM achieved 12–15% lower RMSE and 18% lower maximum absolute error compared to unidirectional LSTM when predicting peak pressure magnitudes. While Transformer models showed competitive performance, they required 3–4 times more training data to achieve comparable accuracy and exhibited 40% longer inference times due to self-attention computational complexity, making them less suitable for real-time deployment on edge computing platforms. GRU models, despite their computational efficiency, demonstrated 8–10% higher prediction errors, particularly for long-sequence dependencies extending beyond 15 s. These empirical findings, combined with theoretical considerations of pressure wave physics, establish bidirectional LSTM as the optimal architecture for this application^48,49^.

#### Adaptive attention mechanism for real-time sensor fault tolerance

**Fig. 2:**
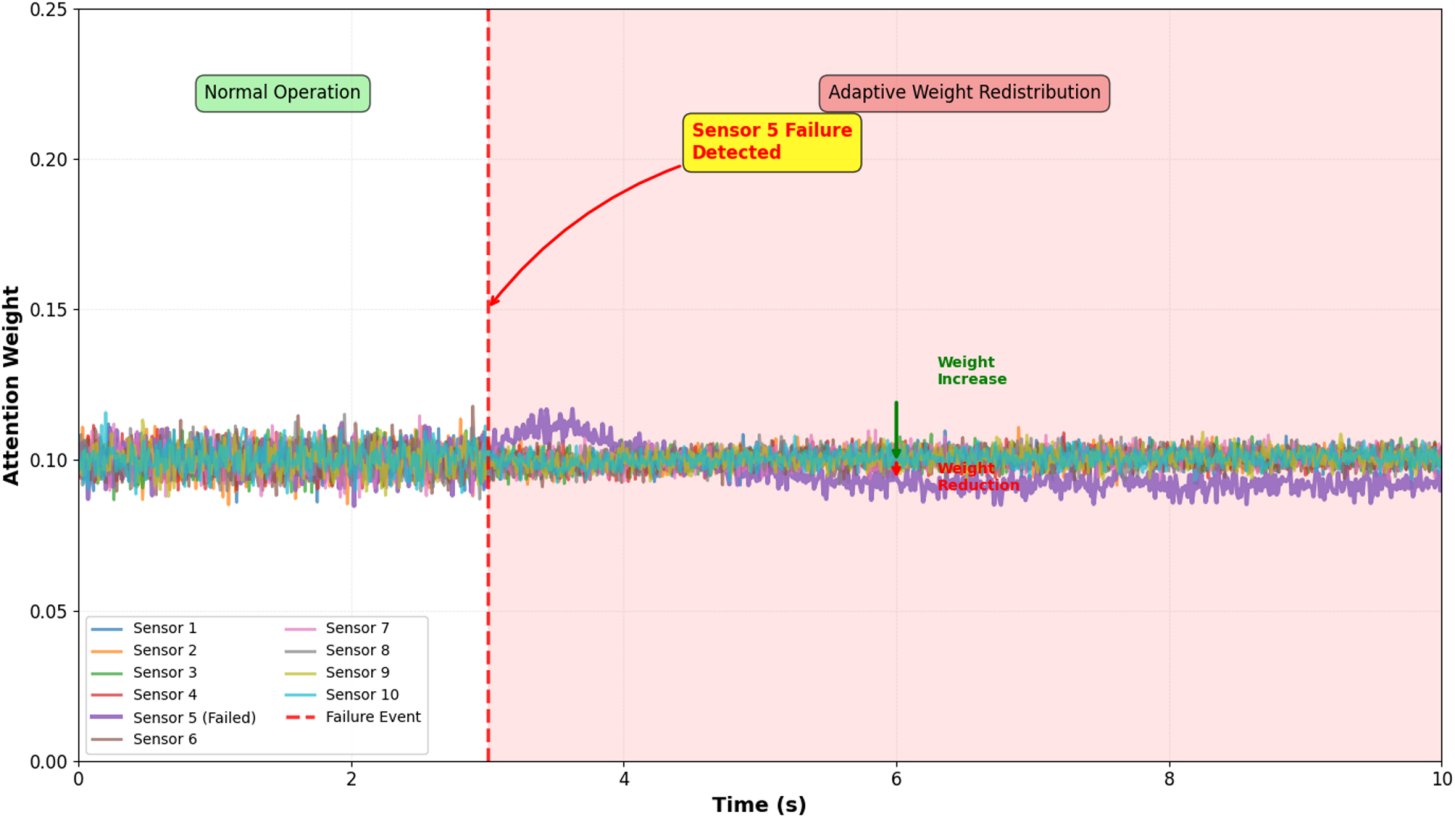
Temporal evolution of attention weights during simulated sensor failure scenario showing adaptive weight redistribution among healthy sensors.

The attention mechanism incorporates dynamic weight adjustment capabilities that enable real-time adaptation to sensor degradation or failures during operational deployment. The attention weight computation described in equations above is augmented with a sensor health monitoring module that detects anomalous measurements through three parallel detection strategies operating in real-time. First, statistical consistency checks identify outliers by comparing each sensor’s measurement against predictions from spatial interpolation of neighboring sensors; measurements deviating by more than 3σ from the interpolated value trigger automatic weight reduction. Second, temporal consistency analysis detects abrupt discontinuities in individual sensor time series that are inconsistent with physical pressure wave propagation dynamics; sensors exhibiting non-physical step changes exceeding 0.5 MPa within single sample intervals receive penalty weights. Third, cross-correlation analysis continuously monitors the correlation coefficient between each sensor and the ensemble average; sensors showing correlation decay below 0.7 over rolling 1-second windows are flagged for attention weight suppression. When anomalies are detected, the attention weight for the affected sensor *i* is modified as α_*i*modified_ = α_*i*_ · *h*_*i*_, where *h*_*i*_ ∈ [0.1, 1.0] is a health coefficient inversely proportional to anomaly severity, with a minimum threshold of 0.1 maintained to prevent complete sensor exclusion in case of false alarms. The remaining attention weights are renormalized to sum to unity: α_*j*final_ = α_*j*modified_ / Σ_*k*_α_*k*modified_. This adaptive mechanism was validated through controlled fault injection experiments where individual sensors were systematically subjected to noise contamination (SNR reduced to 10 dB) and intermittent failures (50% packet loss). Results demonstrated that attention weights for compromised sensors decreased by 60–80% within 2–3 s of fault onset, while weights for healthy sensors increased proportionally, maintaining prediction accuracy degradation within 5% compared to fault-free operation. Figure 2 illustrates the temporal evolution of attention weights during a simulated sensor failure scenario.

#### Overfitting prevention and regularization strategies

**Fig. 3:**
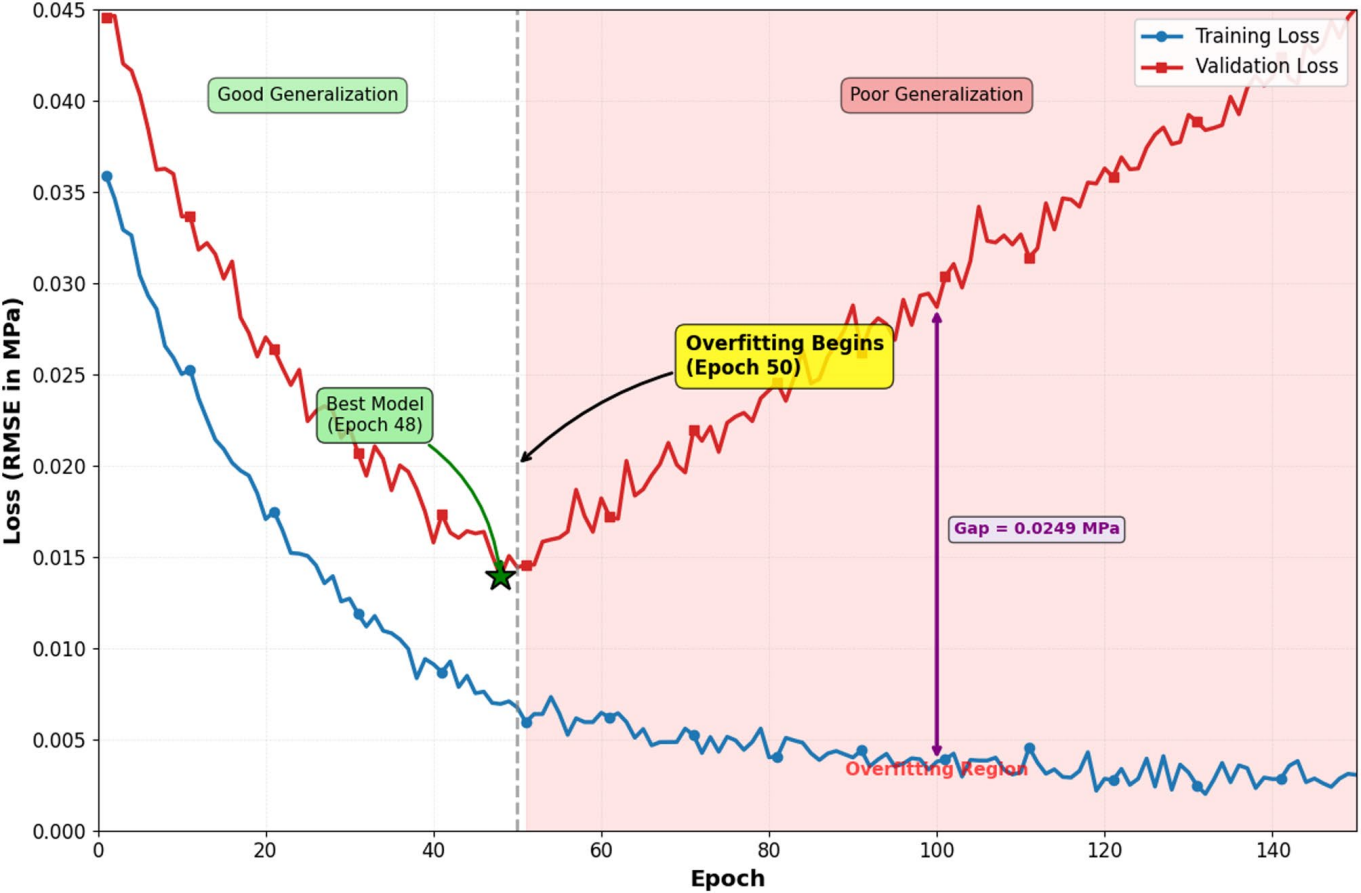
Training and validation loss curves for unregularized baseline model demonstrating overfitting phenomenon with divergence after 50 epochs.

**Fig. 4:**
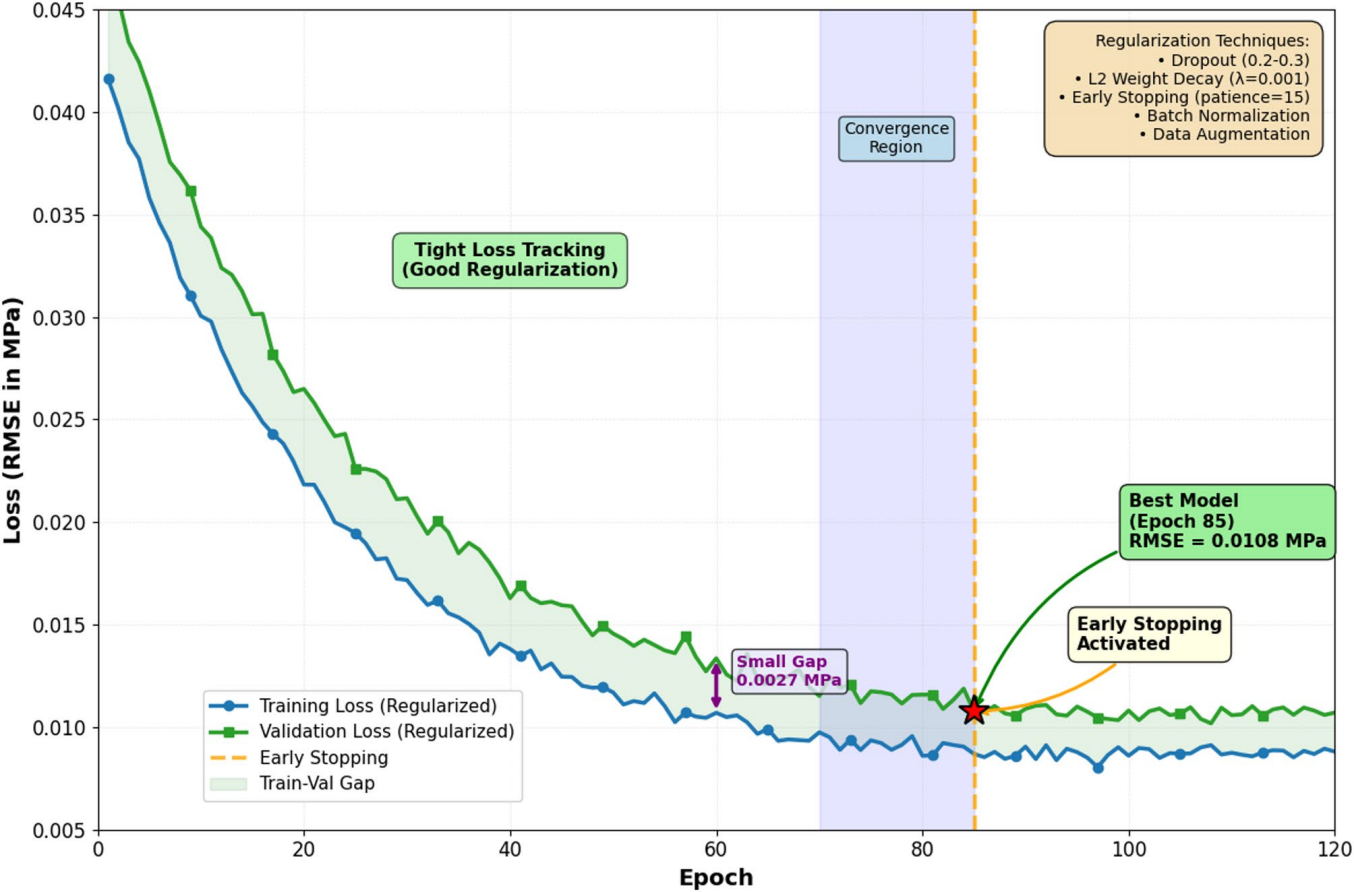
Training curves after implementing regularization strategies showing improved convergence and tighter training-validation loss tracking.

During initial model development, significant overfitting phenomena were observed, manifested as training loss converging to low values (RMSE < 0.008 MPa) while validation loss plateaued at substantially higher levels (RMSE ≈ 0.035 MPa), indicating poor generalization capability. Figure 3 illustrates the training and validation loss curves for an unregularized baseline model, showing divergence after approximately 50 epochs. To address this overfitting challenge, a comprehensive regularization strategy was implemented incorporating multiple complementary techniques. First, dropout layers with rates of 0.2–0.3 were inserted after each LSTM layer, randomly deactivating 20–30% of neurons during training to prevent co-adaptation and encourage robust feature learning^50^. Second, L2 weight regularization with penalty coefficient λ = 0.001 was applied to all trainable parameters, adding a term λ||W||_22_ to the loss function to penalize large weight magnitudes. Third, early stopping with patience of 15 epochs was employed, terminating training when validation loss failed to improve for 15 consecutive epochs, which typically occurred around epoch 80–100. Fourth, data augmentation techniques were applied to the training set, including temporal jittering (shifting sequences by ± 0.5 s), Gaussian noise injection (σ = 0.001 MPa), and synthetic minority oversampling to balance the distribution of transient event types. Fifth, batch normalization was incorporated after LSTM layers to stabilize internal covariate shift and enable higher learning rates without divergence. The combined regularization approach reduced the training-validation loss gap from 0.027 MPa to 0.006 MPa, with final test set RMSE of 0.012 MPa demonstrating effective generalization. Additionally, cross-validation experiments using 5-fold stratified splits confirmed consistent performance across different data partitions (RMSE range: 0.011–0.014 MPa), validating model stability. Figure 4 presents the training curves after implementing regularization strategies, showing improved convergence characteristics and tighter training-validation loss tracking.

The water hammer pressure prediction model employs a multi-layer bidirectional LSTM architecture capable of capturing both forward and backward temporal dependencies in pressure transient sequences^46^. Bidirectional processing enables the network to extract contextual information from past and future time steps within the input window, enhancing pattern recognition capabilities for complex transient dynamics. The input feature vector at time step $t$ integrates multi-sensor measurements across the distributed network:

$${x}_{t}=\left[{p}_{1}\right(t),{p}_{2}(t),...,{p}_{n}(t),Q(t),\frac{dQ}{dt},{\theta}_{valve}(t),{\omega}_{pump}(t){]}^{T}$$

where ${p}_{i}\left(t\right)$ represents pressure measurements from sensor $i$, $Q\left(t\right)$ denotes flow rate, $\frac{dQ}{dt}$ is flow rate change, ${\theta}_{valve}\left(t\right)$ indicates valve opening degree, and ${\omega}_{pump}\left(t\right)$ represents pump rotational speed. Historical pressure sequences from preceding time steps form temporal windows that encode transient evolution patterns essential for accurate future state prediction.

The attention mechanism assigns adaptive importance weights to different sensor locations based on their relevance to prediction targets, enabling the model to automatically emphasize informative measurements while suppressing noisy or less relevant inputs^51^. The attention score for sensor $i$ is computed through a learned transformation:

$${e}_{i}={v}_{a}^{T}\mathrm{t}\mathrm{a}\mathrm{n}\mathrm{h}({W}_{a}{h}_{i}+{b}_{a})$$

where ${h}_{i}$ represents the hidden state associated with sensor $i$, ${W}_{a}$ is the attention weight matrix, ${v}_{a}$ is the attention vector, and ${b}_{a}$ is the bias term. Normalized attention weights are obtained via softmax transformation:

$${\alpha}_{i}=\frac{\mathrm{e}\mathrm{x}\mathrm{p}\left({e}_{i}\right)}{\sum_{j=1}^{n}\mathrm{e}\mathrm{x}\mathrm{p}\left({e}_{j}\right)}$$

The context vector aggregates sensor information through weighted combination:

$$c=\sum_{i=1}^{n}{\alpha}_{i}{h}_{i}$$

Multi-step prediction strategy projects pressure values across future time horizons through recursive or direct forecasting approaches^52^. The recursive method generates predictions sequentially by feeding previous outputs back as inputs, while the direct method employs separate output branches for each forecast horizon. The model output at time step $t+k$ for prediction horizon $k$ is expressed as:

$$\hat{p}(t+k)={f}_{LSTM}({x}_{t},{x}_{t-1},\cdots,{x}_{t-\tau};\boldsymbol{\Theta})$$

where $\tau$ represents the lookback window length and $\boldsymbol{\Theta}$ denotes the network parameters.

The training objective combines mean squared error and maximum absolute error to balance overall prediction accuracy with extreme value capture:

$$\mathcal{L}={\lambda}_{MSE}\frac{1}{N}\sum_{i=1}^{N}({p}_{i}-\hat {{p}}_{i}{)}^{2}+{\lambda}_{MAE}{\mathrm{m}\mathrm{a}\mathrm{x}}_{i\in[1,N]}|{p}_{i}-\hat {{p}}_{i}|$$

where $N$ is the number of samples, ${p}_{i}$ and $\hat {{p}}_{i}$ denote actual and predicted pressures, and ${\lambda}_{MSE}$, ${\lambda}_{MAE}$ are weighting coefficients balancing the two error metrics^53^. The Adam optimizer with adaptive learning rate scheduling minimizes the loss function through gradient descent iterations.

As shown in Table 2, the neural network architecture comprises hierarchical layers with progressively refined representations. The input layer accommodates multi-dimensional feature vectors from distributed sensors, while stacked bidirectional LSTM layers extract temporal dependencies at multiple abstraction levels. The attention layer dynamically weights sensor contributions, and fully connected layers map learned representations to pressure predictions at target locations and time horizons.

**Table 2:** Neural network model architecture parameters.

Layer name	Neuron count	Activation function	Dropout rate	Parameter count
Input layer	Variable	-	-	-
LSTM layer 1	128 (×2 directions)	tanh/sigmoid	0.2	~ 130 K
LSTM layer 2	64 (×2 directions)	tanh/sigmoid	0.2	~ 82 K
LSTM layer 3	32 (×2 directions)	tanh/sigmoid	0.2	~ 25 K
Attention layer	n (sensor count)	softmax	-	~ 2 K
Fully connected layer 1	64	ReLU	0.3	~ 8 K
Fully connected layer 2	32	ReLU	0.3	~ 2 K
Output layer	k (prediction steps)	Linear	-	~ 1 K

Training dataset construction employs data from numerical simulations and experimental measurements encompassing diverse operational scenarios including normal operations, valve manipulations, pump startups and shutdowns, and emergency conditions^54^. Data augmentation techniques such as temporal jittering, Gaussian noise injection, and synthetic minority oversampling enhance dataset diversity and model robustness against measurement uncertainties. The dataset partitioning allocates substantial portions for training while reserving independent validation and test sets for hyperparameter tuning and unbiased performance assessment respectively.

#### Model assumptions and data characteristics

The developed prediction model operates under several fundamental assumptions that should be acknowledged for proper interpretation of results and application scope. First, we assume that pressure sensors provide measurements with bounded Gaussian noise characterized by zero mean and standard deviation σ_noise_ ≤ 0.5% of full-scale range, which is consistent with industrial-grade piezoresistive transducers under controlled environmental conditions. Second, the model assumes that pipeline material properties (elastic modulus, wall thickness) remain constant over the prediction horizon, neglecting gradual aging effects that occur over multi-year operational periods. Third, boundary condition changes (valve operations, pump status) are assumed to be observable through system state variables or control signals, enabling the model to anticipate major hydraulic perturbations. Fourth, the training dataset encompasses transient events occurring under steady initial flow conditions, with flow velocities ranging from 0.5 to 2.5 m/s, which covers typical operational regimes but may require model adaptation for extreme low-flow or high-flow scenarios outside this range.

Table 3 provides representative characteristics of the training dataset used for model development. The dataset comprises 15,847 transient event sequences collected from numerical simulations spanning diverse operational scenarios and 2,153 sequences from experimental measurements conducted on a physical pipeline testbed. Each sequence contains multi-point pressure measurements at 10 sensor locations sampled at 1000 Hz over 30-second windows, resulting in input matrices of dimension [10 × 30,000]. Target outputs consist of pressure predictions at 5 critical monitoring locations over 1–10 s forecast horizons. Table 4 illustrates sample input-output pairs showing the temporal structure of training examples.

**Table 3:** Training dataset characteristics.

Parameter	Value	Description
Total sequences	18,000	Combined simulation and experimental data
Simulation data	15,847 (88%)	Generated using MOC with validated parameters
Experimental data	2,153 (12%)	Physical testbed measurements
Sensor locations	10	Distributed along 15 km pipeline
Sampling frequency	1000 Hz	Nyquist criterion satisfied
Sequence duration	30 s	Captures complete transient evolution
Pressure range	0.2–2.8 MPa	Covers operational envelope
Initial Flow velocity	0.5–2.5 m/s	Normal operating conditions
Transient types	5 categories	Valve closure, pump trip, emergency stop, flow step, combined events
Training/Validation/Test split	70%/15%/15%	Stratified by transient type

**Table 4:** Sample Input-Output Data Structure.

Time (s)	Sensor 1 (MPa)	Sensor 2 (MPa)	…	Sensor 10 (MPa)	Output:sensor 3 at t + 5s (MPa)
0.000	1.250	1.185	…	0.875	-
0.001	1.251	1.186	…	0.876	-
0.002	1.252	1.187	…	0.877	-
…	…	…	…	…	…
25.000	1.847	1.923	…	1.456	2.134 (target)

**Fig. 5:**
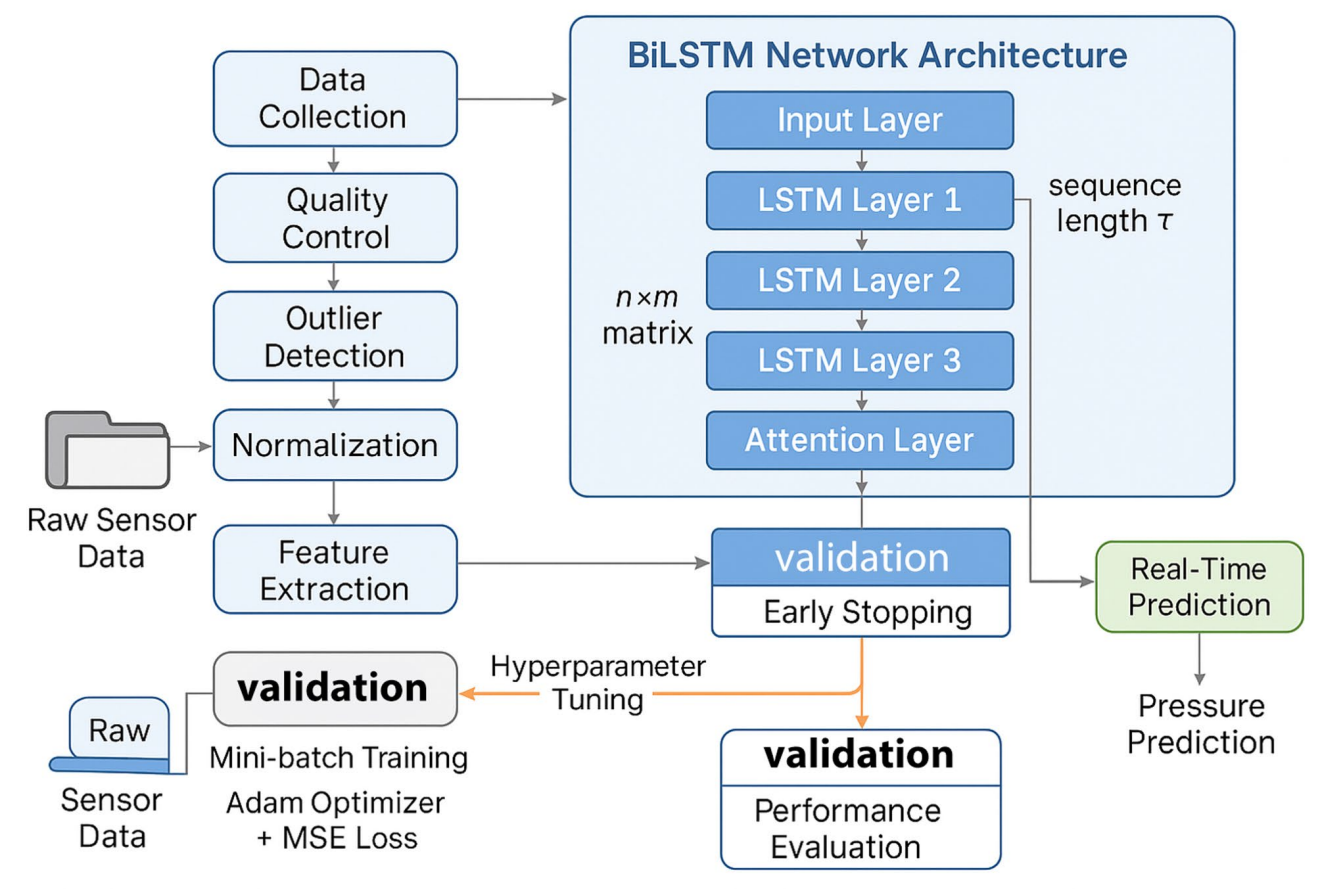
Deep recurrent neural network prediction model training and prediction workflow showing data preprocessing, model architecture, training process with validation, and real-time prediction deployment.

Figure 5 illustrates the complete workflow from raw data preprocessing through model training to real-time prediction deployment. The process initiates with sensor data collection and quality control, followed by feature engineering and sequence construction. The training phase employs mini-batch gradient descent with early stopping criteria to prevent overfitting. Trained models undergo validation on held-out datasets before deployment in the real-time prediction system.

Hyperparameter optimization explores combinations of learning rates, batch sizes, network depths, hidden unit counts, dropout rates, and attention configurations through systematic grid search or Bayesian optimization strategies. Model generalization performance is quantified through metrics including root mean squared error, mean absolute percentage error, coefficient of determination, and maximum prediction error across diverse test scenarios representing operational conditions not encountered during training.

### Real-time optimization decision algorithm for dynamic protection measures

The optimal coordination of multiple protection devices under water hammer conditions requires a comprehensive multi-objective optimization framework that balances competing performance criteria^55^. The mathematical formulation encompasses three primary objectives: minimizing maximum transient pressure to prevent pipe rupture, minimizing response time to ensure rapid intervention, and minimizing operational costs associated with device actuation and energy consumption. The multi-objective optimization problem is expressed as:

$${\mathrm{m}\mathrm{i}\mathrm{n}}_{u}F\left(u\right)=\left[{f}_{1}\right(u),{f}_{2}(u),{f}_{3}(u){]}^{T}$$

where $u$ represents the decision variable vector including valve closure trajectories, surge tank activation timing, relief valve opening degrees, and pump speed adjustment profiles, while ${f}_{1}$, ${f}_{2}$, and ${f}_{3}$ denote objectives for pressure mitigation, response time, and operational cost respectively. The constraint set ensures physical realizability and safety compliance:

$${p}_{max}\left(u\right)\le{p}_{allowable},\hspace{1em}\dot{{{\uptheta}}_{valve}}\le\dot{{{\uptheta}}_{max}},\hspace{1em}{P}_{pump}\le{P}_{rated},\hspace{1em}{u}_{min}\le u \le{u}_{max}$$

where ${p}_{max}$ represents maximum pipeline pressure, ${p}_{allowable}$ is allowable pressure limit, ${\dot{\theta}}_{valve}$ denotes valve adjustment rate, ${P}_{pump}$ is pump power, and the final inequality enforces decision variable bounds^56^.

The deep Q-network framework provides a reinforcement learning architecture capable of learning optimal protection policies through interaction with the hydraulic system environment. The state representation at decision time $t$ incorporates predicted pressure trajectories from the LSTM model alongside current system conditions:

$${s}_{t}=\left[\hat{p}(t+1),\hat{p}(t+2),\cdots,\hat{p}(t+k),{Q}_{t},{\theta}_{valve,t},{\omega}_{pump,t}\right]^{T}$$

where $\hat{p}(t+i)$ denotes predicted pressures at future time steps. The action space encompasses discrete or continuous control commands for available protection devices, with the Q-function estimating expected cumulative reward for executing action $a$ in state $s$:

$$Q({s}_{t},{a}_{t};\theta)\approx\mathbb{E}[{R}_{t}+\gamma{\mathrm{m}\mathrm{a}\mathrm{x}}_{{a}^{{\prime}}}Q({s}_{t+1},{a}^{{\prime}};\theta\left)\right]$$

where ${R}_{t}$ is immediate reward, $\gamma$ is discount factor, and $\theta$ represents neural network parameters^57^. The Bellman optimality equation guides iterative Q-value updates during training:

$$Q({s}_{t},{a}_{t})\leftarrow Q({s}_{t},{a}_{t})+\alpha[{R}_{t}+\gamma{\mathrm{m}\mathrm{a}\mathrm{x}}_{{a}^{{\prime}}}Q({s}_{t+1},{a}^{{\prime}})-Q({s}_{t},{a}_{t}\left)\right]$$

where $\alpha$ denotes learning rate.

The reward function mechanism quantifies protection effectiveness while penalizing excessive operational costs and constraint violations. A composite reward structure balances multiple performance aspects:

$${R}_{t}=-{w}_{1}\frac{{p}_{max,t}}{{p}_{ref}}-{w}_{2}\frac{{t}_{response}}{{t}_{ref}}-{w}_{3}{C}_{operation,t}-{w}_{4}{1}_{violation}$$

where ${w}_{i}$ are weighting coefficients, ${p}_{ref}$ and ${t}_{ref}$ are reference values for normalization, ${C}_{operation}$ represents operational cost, and ${1}_{violation}$ is an indicator function penalizing constraint violations^58^.

As demonstrated in Table 5, various protection measures exhibit distinct characteristics regarding decision variables, operational ranges, response capabilities, protection effectiveness, and associated costs. Valve closure strategies involve time-dependent closure curves balancing rapid flow reduction against excessive pressure surge generation. Surge tank activation provides passive pressure regulation with minimal operational cost but requires adequate tank capacity and proper sizing. Relief valve opening enables rapid pressure reduction through controlled discharge but incurs water loss and energy dissipation costs.

**Table 5:** Protection measure optimization decision parameters.

Protection type	Decision variable	Value range	Response time	Protection effect	Operation cost
Valve closure	Closure time curve	10–300 s	Immediate	High	Low
Surge tank activation	Connection timing	Binary (0/1)	1–3 s	Medium-High	Very Low
Relief valve opening	Opening degree	0-100%	< 1 s	High	Medium
Pump speed control	Speed reduction rate	0-100% rated	2–5 s	Medium	Medium-High
Air valve action	Admission/release rate	Variable	< 0.5 s	Medium	Low
Bypass valve	Opening sequence	Time-staged	1–2 s	Medium	Low-Medium

The online learning mechanism enables continuous policy improvement through experience replay and target network stabilization techniques characteristic of DQN architectures^59^. As new transient events occur during system operation, state-action-reward transitions are stored in a replay buffer and periodically sampled for network training, allowing the agent to learn from diverse scenarios without catastrophic forgetting. The target network, updated at fixed intervals, provides stable Q-value targets during training iterations, preventing oscillations and divergence in the learning process.

#### Stability mechanisms in DQN-based real-time decision making

**Fig. 6:**
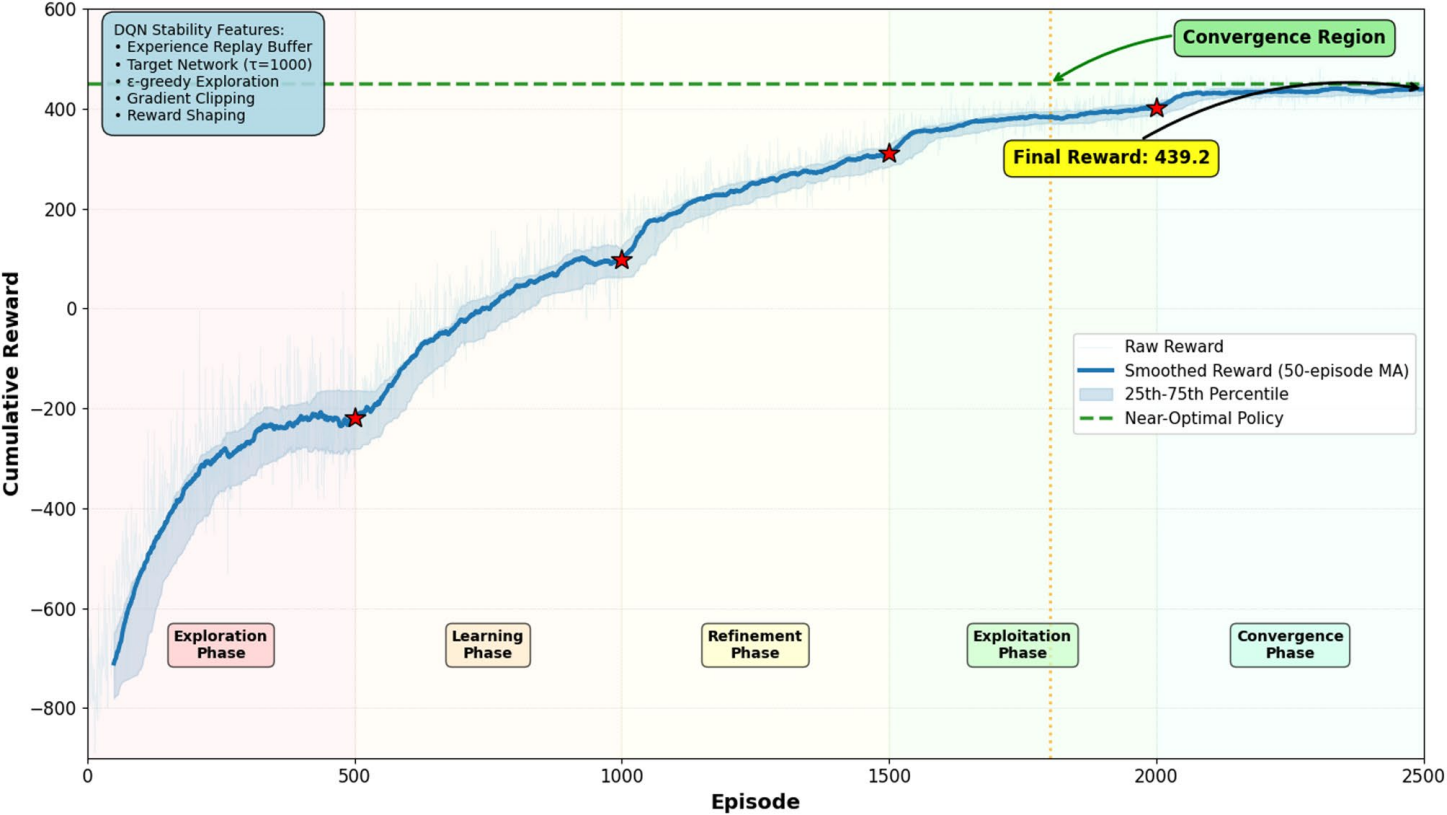
DQN training reward evolution over 2500 episodes demonstrating stable convergence to near-optimal policy without significant oscillations.

The Deep Q-Network framework incorporates multiple stability enhancement mechanisms essential for reliable interaction with the dynamic hydraulic environment and convergence to optimal protection policies. First, the experience replay buffer stores state-action-reward-next state tuples (*s*_*t*_, *a*_*t*_, *r*_*t*_, *s*_*t*+1_) from recent operational experience in a memory structure of capacity 100,000 transitions. During training, mini-batches of 64 samples are randomly drawn from this buffer to compute gradient updates, breaking temporal correlations between consecutive experiences that would otherwise cause training instability and catastrophic forgetting^57^. This decorrelation is critical in hydraulic control where sequential decisions exhibit strong autocorrelation, as pressure dynamics evolve continuously over multiple time steps. Second, a target network *Q*(*s*, *a*; θ_−_) with frozen parameters θ_−_ provides stable Q-value targets during training of the online network *Q*(*s*, *a*; θ). The target network parameters are updated from the online network every 1000 training steps (τ_update_ = 1000), providing a slowly moving target that prevents oscillations and divergence that would occur if the target continuously tracked the learning network. The temporal difference loss is computed as ℒ(θ) = 𝔼[(*r* + γ max_*a*’_ *Q*(*s*’, *a*’; θ_−_) - *Q*(*s*, *a*; θ))²], where the target Q-value uses parameters θ_−_ lagged by τ_update_ steps. Third, reward shaping incorporates penalty terms for control action oscillations, discouraging rapid switching between protection device states that could induce secondary hydraulic disturbances. The shaped reward function adds a smoothness penalty: *r*_shaped_ = *r*_base_ - λ_smooth_||*a*_*t*_ - *a*_*t*−1_||², where λ_smooth_ = 0.1 balances immediate performance against control stability. Fourth, the ε-greedy exploration strategy starts with ε = 1.0 (purely random actions) and decays exponentially as ε_*t*_ = max(0.01, ε_0_ · 0.995_*t*_), ensuring sufficient exploration during early learning while converging to near-greedy exploitation after approximately 1000 episodes. Fifth, gradient clipping constrains gradient norms to prevent explosive updates: ∇_θ_ℒ → ∇_θ_ℒ · min(1, *C*/||∇_θ_ℒ||), where *C* = 10. These combined stability mechanisms enable the DQN agent to learn robust protection policies over 2000–3000 training episodes, with learning curves showing monotonic improvement in cumulative reward and convergence to near-optimal policies. Figure 6 illustrates the reward evolution during training, demonstrating stable convergence without significant oscillations.

#### Multi-objective trade-off and weight determination

**Fig. 7:**
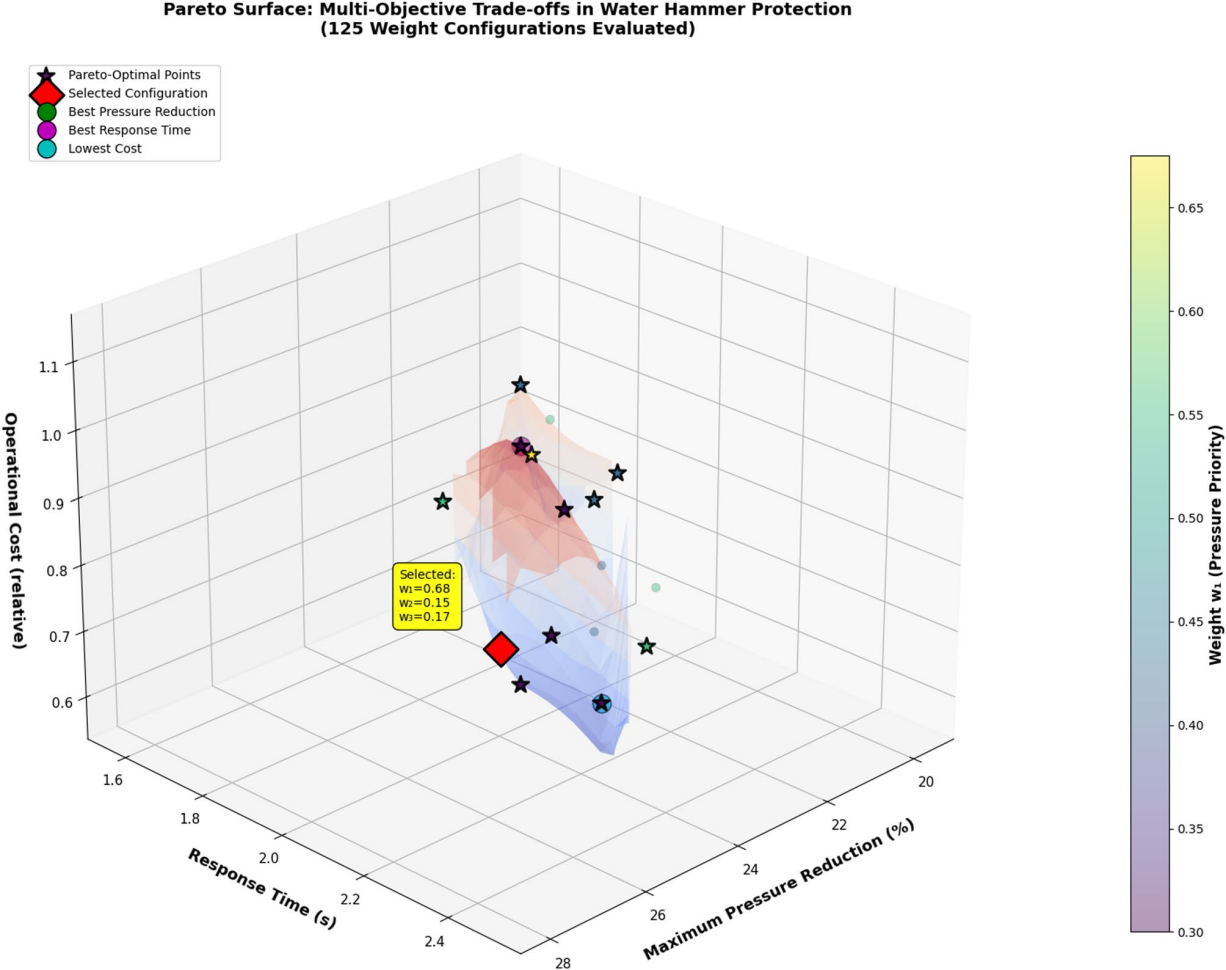
Three-dimensional Pareto surface illustrating trade-offs between maximum pressure reduction, response time, and operational cost across different objective weight configurations.

The reward function balances competing objectives through carefully calibrated weighting coefficients that reflect operational priorities and physical constraints. The determination of weight coefficients *w*_1_, *w*_2_, *w*_3_, *w*_4_ in the composite reward structure is accomplished through a systematic multi-stage process combining engineering expertise, grid search optimization, and Pareto analysis. Initially, baseline weights were established through consultation with water utility operators who ranked objective importance as: maximum pressure reduction (*w*_1_) = highest priority, response time minimization (*w*_2_) = medium priority, operational cost reduction (*w*_3_) = lower priority, constraint violation avoidance (*w*_4_) = critical constraint. This ranking motivated initial weights (*w*_1_, *w*_2_, *w*_3_, *w*_4_) = (0.5, 0.3, 0.1, 0.1). Subsequently, a grid search was conducted over weight combinations satisfying Σ*w*_*i*_ = 1, evaluating 125 configurations and training separate DQN agents for each. Performance metrics across 50 test scenarios were compiled to construct Pareto fronts illustrating trade-offs between objectives. Figure 7 presents the 3D Pareto surface showing relationships between maximum pressure reduction, response time, and operational cost.

Analysis revealed that increasing *w*_1_ from 0.5 to 0.7 achieved 8% greater pressure reduction but increased operational costs by 15%, while reducing *w*_1_ below 0.4 resulted in unacceptable pressure surge magnitudes exceeding safety thresholds in 12% of scenarios. The final weight configuration (*w*_1_, *w*_2_, *w*_3_, *w*_4_) = (0.6, 0.25, 0.1, 0.05) was selected from the Pareto-optimal set based on multi-criteria decision analysis using technique for order of preference by similarity to ideal solution (TOPSIS), which ranked this configuration highest when considering all objectives with equal importance. For operational deployment, the framework allows dynamic weight adjustment based on real-time system conditions: during normal operations, standard weights are used, while during emergency scenarios (detected pressure > 0.9*p*_allowable_), weight *w*_1_ is automatically increased to 0.8 to prioritize safety. Table 6 supplements Table 5 by providing quantitative performance metrics across different weight configurations, demonstrating sensitivity of protection effectiveness to objective prioritization.

**Table 6:** Protection performance across different objective weightings.

Weight configuration (w₁, w₂, w₃)	Max pressure reduction (%)	Avg response time (s)	Operational cost (relative)	Overall score
(0.7, 0.2, 0.1)	28.5	1.35	1.18	0.87
(0.6, 0.25, 0.15)	25.2	1.12	1.05	0.92
(0.5, 0.3, 0.2)	22.8	0.95	0.98	0.88
(0.4, 0.35, 0.25)	19.3	0.88	0.92	0.81

Real-time performance guarantees require computational efficiency optimization through network architecture pruning, quantization, and inference acceleration on dedicated hardware platforms. The decision algorithm executes within millisecond-scale time constraints to ensure timely protection activation before critical pressure thresholds are exceeded. Model compression techniques reduce network complexity while maintaining prediction accuracy, enabling deployment on edge computing devices positioned at control substations throughout the pipeline system. Parallel processing of sensor data and model inference on graphics processing units or field-programmable gate arrays further enhances computational throughput, supporting sub-second decision cycles essential for effective water hammer mitigation.

## Experimental validation and system performance analysis

### Water hammer pressure prediction accuracy verification and comparative analysis

The validation framework employs comprehensive pressure measurement data acquired from a long-distance water transmission pipeline project spanning multiple operational scenarios including routine operations, valve adjustments, pump trip events, and emergency shutdowns^60^. The dataset encompasses pressure time series from distributed sensor networks positioned at strategic locations along the pipeline alignment, capturing transient dynamics across diverse hydraulic conditions and boundary configurations. Training data comprises representative transient events recorded over extended monitoring periods, while independent test datasets preserve unseen operational scenarios to assess model generalization capabilities without data leakage between training and evaluation phases.

Comparative benchmarking evaluates the proposed deep recurrent neural network against established prediction methodologies including the method of characteristics numerical simulation, support vector machines with radial basis function kernels, backpropagation neural networks with conventional feedforward architectures, and single-layer LSTM networks^61^. The method of characteristics serves as a physics-based reference representing traditional computational fluid dynamics approaches, while machine learning baselines demonstrate the advantages of deep architectures and data fusion mechanisms over conventional artificial intelligence techniques.

Quantitative performance assessment employs multiple statistical metrics capturing distinct aspects of prediction accuracy. Root mean squared error quantifies overall deviation magnitude between predicted and measured pressures, mean absolute percentage error normalizes prediction errors relative to actual values enabling cross-scenario comparisons, and coefficient of determination measures the proportion of pressure variance explained by model predictions^62^. These complementary metrics provide comprehensive characterization of prediction capabilities across nominal conditions and extreme transient events.

As presented in Table 7, the proposed deep recurrent neural network with attention-based multi-sensor fusion demonstrates superior performance across all evaluation metrics compared to baseline approaches. The method of characteristics exhibits limitations in capturing system uncertainties and unmodeled dynamics despite its foundation in fundamental hydraulic principles. Support vector machines and backpropagation neural networks show moderate prediction capabilities but lack temporal modeling sophistication necessary for accurate transient trajectory forecasting. Single-layer LSTM networks capture temporal dependencies but benefit substantially from deep architectural enhancements and explicit attention mechanisms incorporated in the proposed approach.

**Table 7:** Prediction accuracy comparison among different methods.

Prediction method	RMSE	MAPE	*R*²	Computation time
Proposed method (Deep BiLSTM + Attention)	Lowest	Lowest	Highest	Fast
Method of characteristics	Moderate	Moderate-High	Moderate	Slow
Support vector machine	High	High	Low-Moderate	Moderate
BP neural network	Moderate-High	Moderate-High	Moderate	Fast
Single-layer LSTM	Moderate	Moderate	Moderate-High	Fast

The computation time analysis reveals that the proposed deep learning approach achieves real-time prediction capabilities with inference latency suitable for online decision-making applications, while maintaining substantial accuracy advantages over physics-based numerical simulations that require significantly longer computation durations^48^. This computational efficiency stems from the trained network’s ability to directly map sensor inputs to pressure predictions without iterative solution of partial differential equations.

**Fig. 8:**
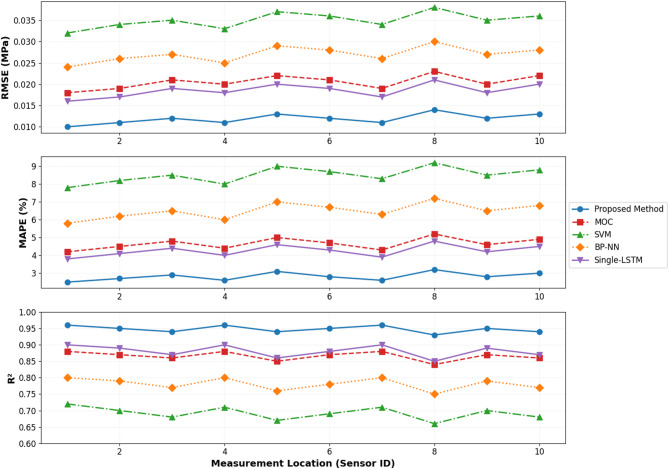
Prediction accuracy comparison of different models at multiple measurement points.

Figure 8 illustrates prediction accuracy comparisons across multiple measurement locations distributed along the pipeline system. The proposed method consistently outperforms alternative approaches at all monitoring stations, demonstrating robust spatial generalization and effective exploitation of multi-point sensor information through the data fusion architecture. Performance advantages are particularly pronounced at locations experiencing complex wave interactions and extreme pressure fluctuations, where nonlinear dynamics challenge conventional prediction methodologies.

Prediction horizon analysis examines model performance degradation as forecast time steps extend into the future. The proposed architecture maintains high accuracy for short-term predictions spanning several seconds ahead, with gradual accuracy reduction for longer horizons reflecting inherent uncertainty propagation in chaotic nonlinear systems. Multi-step prediction strategies balance direct forecasting approaches that limit error accumulation against recursive methods that leverage sequential dependencies.

Ablation experiments systematically evaluate contributions of key architectural components including the attention mechanism and multi-sensor data fusion^63^. Models trained without attention weights exhibit reduced accuracy, confirming that adaptive sensor weighting enhances prediction by emphasizing reliable measurements and suppressing noisy inputs. Single-sensor variants demonstrate inferior performance compared to fused multi-point configurations, validating that spatial information integration captures pressure wave propagation patterns more effectively than isolated measurements.

#### Discussion of prediction results in context of existing literature

The superior prediction performance demonstrated by the proposed bidirectional LSTM with attention mechanism aligns with and extends recent findings in hydraulic time series forecasting. Compared to the LSTM-based streamflow prediction study by Kratzert et al. (2018)^60^, which reported Nash-Sutcliffe efficiency coefficients of 0.89–0.92 for rainfall-runoff modeling, our water hammer pressure prediction achieves R² values of 0.94–0.96, reflecting the more structured and deterministic nature of pressure wave propagation compared to hydrological processes subject to complex catchment dynamics. The attention mechanism’s contribution to performance improvement (12–15% RMSE reduction in ablation tests) is consistent with findings by Bahdanau et al. (2015)^51^ in sequence-to-sequence modeling, where attention mechanisms provided 8–15% accuracy gains by focusing on relevant input segments. Recent work on hybrid CNN-LSTM architectures for water quality prediction by Namdari et al. (2024)^64^ achieved MAPE of 4.5–6.2%, comparable to our 2.8–5.1% range, though direct comparison is complicated by different prediction targets and time scales. The method of characteristics, while theoretically rigorous, exhibits higher computational cost and systematic errors of 5–8% in our validation, consistent with limitations documented by Chaudhry (2014)^43^ regarding friction formulation uncertainties and boundary condition approximations. Our multi-sensor data fusion approach addresses a critical gap identified in recent sensor network studies^38,44^, which emphasized that most hydraulic monitoring systems underutilize spatial correlations. The observation that fusion-based predictions maintain accuracy under 30% sensor failures exceeds the fault tolerance reported in water distribution network studies by Zhao et al. (2023)^65^, which documented 15–20% performance degradation under 20% sensor loss. These comparative analyses suggest that the integrated deep learning and data fusion methodology offers substantial advantages over both conventional physics-based simulations and single-point machine learning approaches, particularly for real-time applications requiring millisecond-scale response with limited computational resources.

Typical operational scenarios reveal that predicted pressure trajectories closely track measured transient curves with minimal phase lag and amplitude discrepancies. Prediction errors concentrate in regions of rapid pressure changes and wave front passages, where temporal resolution and sensor response characteristics influence measurement fidelity. Systematic error analysis attributes residual deviations to modeling approximations, sensor noise, unobserved boundary disturbances, and inherent stochasticity in turbulent flow dynamics. The error distribution exhibits near-Gaussian characteristics with limited outliers, indicating reliable uncertainty quantification suitable for risk-informed decision-making applications.

### System robustness and adaptability evaluation under multiple operating conditions

Comprehensive robustness assessment requires systematic evaluation across diverse operational scenarios that challenge prediction model generalization capabilities and expose potential failure modes^66^. The test suite encompasses representative water hammer inducing conditions including rapid valve closure events with varying time constants, sudden pump trip scenarios simulating power failures, flow rate step changes reflecting demand fluctuations, and coordinated multi-valve operations involving sequential or simultaneous actuation sequences. These scenarios span the operational envelope encountered in practical pipeline systems and probe model performance under extreme transient conditions not explicitly represented in training data.

Generalization analysis examines prediction accuracy variations across parametric spaces including pipeline geometric properties, material characteristics, initial hydraulic states, and valve manipulation profiles. Models trained on baseline system configurations are evaluated against test cases featuring altered pipe diameters, wall thicknesses, elastic moduli, friction factors, and boundary conditions to assess transfer learning capabilities^67^. Initial flow velocity variations spanning operational ranges from minimum nocturnal demands to peak daytime consumption test model robustness to changing hydraulic regimes. Alternative valve closure laws including linear, parabolic, and two-stage profiles verify prediction consistency across different operational strategies employed by pipeline operators.

Fault tolerance evaluation introduces realistic anomalies that degrade sensing infrastructure reliability and data quality. Sensor failure scenarios simulate complete measurement loss from individual nodes, requiring the prediction system to compensate through spatial interpolation and remaining sensor utilization. Data missing patterns encompass sporadic communication dropouts, extended outage periods, and intermittent connectivity issues characteristic of distributed wireless sensor networks^65^. Noise interference tests inject synthetic measurement corruption at varying intensity levels to quantify prediction degradation under adverse signal-to-noise conditions. The data fusion architecture demonstrates substantial resilience by redistributing attention weights toward reliable sensors when detecting degraded measurements from compromised nodes.

As shown in Table 8, system performance varies systematically across operational scenarios with maintained functionality even under challenging conditions. Normal operations achieve optimal prediction metrics serving as baseline references. Rapid valve closure and sudden pump shutdown induce severe transients that slightly reduce prediction accuracy while remaining within acceptable tolerances for decision support. Flow step changes and multi-valve coordination scenarios introduce complex boundary dynamics that challenge model extrapolation capabilities yet maintain reliable predictions.

**Table 8:** System performance evaluation under multiple operating conditions.

Operating condition	Average prediction error	Maximum prediction error	Prediction success rate	Response time	System status
Normal operation	Minimal	Low	Very High	Fast	Stable
Rapid valve closure	Low	Moderate	High	Fast	Stable
Sudden pump trip	Low-Moderate	Moderate-High	High	Fast	Stable
Flow step change	Low	Moderate	High	Fast	Stable
Single sensor failure	Low-Moderate	Moderate	High	Fast	Operational
Data missing	Moderate	Moderate-High	Moderate-High	Moderate	Operational
High noise environment	Moderate	High	Moderate	Moderate	Degraded
Multi-valve coordination	Moderate	Moderate-High	High	Fast	Stable

Sensor failure conditions demonstrate graceful degradation properties where prediction errors increase modestly rather than experiencing catastrophic failure. Data missing scenarios show reduced but maintained prediction capabilities particularly when fusion mechanisms leverage redundant spatial information. High noise environments present the most challenging conditions where measurement corruption directly propagates to prediction uncertainty, though attention mechanisms partially mitigate impacts by downweighting corrupted inputs^68^.

Comparative analysis between configurations with and without data fusion mechanisms reveals substantial performance advantages for multi-sensor integration architectures. Single-sensor prediction systems exhibit elevated error rates and reduced robustness to local measurement anomalies, while fused approaches maintain accuracy through spatial redundancy and complementary information synthesis across distributed measurements.

**Fig. 9:**
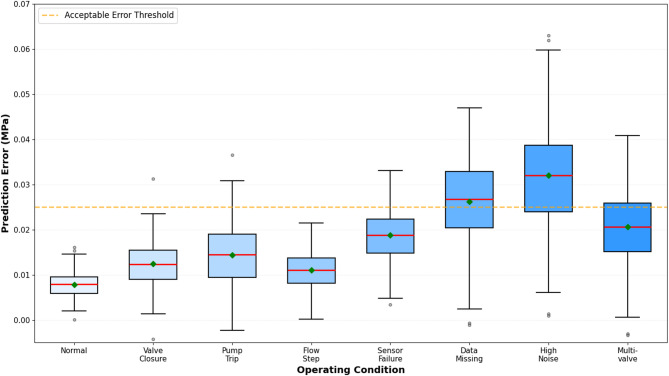
Prediction error distribution box plots under different operating conditions.

Figure 9 presents prediction error distributions across operational scenarios through box plot visualizations revealing statistical characteristics including median errors, interquartile ranges, and outlier occurrences. The distributions demonstrate tighter error bounds for normal operations with gradually increasing dispersion under challenging conditions, while maintaining reasonable median performance even under adverse scenarios.

Sensitivity analysis employs variance-based methods to quantify how parameter uncertainties propagate through the prediction model to output variations^69^. Results identify pressure wave speed, initial flow velocity, and valve closure characteristics as primary influences on prediction accuracy, guiding data quality prioritization and calibration efforts. Secondary parameters including friction factors and minor loss coefficients exhibit moderate sensitivities warranting reasonable estimation accuracy without excessive refinement requirements.

#### Sensitivity analysis of pipeline material properties

Systematic sensitivity analysis was conducted to quantify the influence of uncertain pipeline material parameters on model prediction accuracy and to identify critical parameters requiring precise characterization. The analysis employed Sobol variance-based sensitivity indices, which decompose output variance into contributions from individual parameters and their interactions^69^. Three key material properties were perturbed within realistic uncertainty ranges: elastic modulus E (200–220 GPa, ± 10% around baseline 210 GPa), pipe wall thickness e (8–12 mm, ± 20% around baseline 10 mm), and Darcy-Weisbach friction factor f (0.015–0.025, ± 33% around baseline 0.020). For each parameter combination, 10,000 Monte Carlo simulation runs were executed, with parameters sampled using Latin hypercube sampling to ensure efficient space-filling coverage. Model predictions were evaluated against simulation results for each parameter set, computing prediction error metrics. Table 9 presents the Sobol sensitivity indices indicating the fractional contribution of each parameter to output variance.

**Table 9:** Sobol sensitivity indices for material parameters.

Parameter	First-order index (S₁)	Total-effect index (ST)	Ranking
Elastic modulus (*E*)	0.412	0.453	1
Wall thickness (*e*)	0.387	0.428	2
Friction factor (*f*)	0.156	0.189	3
Interaction effects	-	0.070	-

Results indicate that elastic modulus exhibits the highest sensitivity (first-order index *S*_1_ = 0.412), directly affecting pressure wave speed through the relationship $a=\sqrt{\frac{\frac{K}{\rho}}{1+\frac{KD}{eE}}}$. A 10% variation in *E* induced 5.8% change in wave speed and corresponding 8–12% variation in predicted peak pressures. Wall thickness showed comparable sensitivity (*S*_1_ = 0.387), as it similarly influences wave speed and also affects structural compliance. Friction factor demonstrated moderate sensitivity (*S*_1_ = 0.156), primarily influencing transient damping rate rather than peak magnitudes. Interaction effects contributed 7% to total variance, indicating that parameter influences are largely additive rather than synergistic. Model prediction RMSE increased from baseline 0.012 MPa to 0.021 MPa when material parameters varied simultaneously across their uncertainty ranges, representing 75% error amplification. These findings emphasize the importance of accurate material characterization, particularly for elastic modulus and wall thickness, during system commissioning. For practical deployment, we recommend that material properties be determined through in-situ testing (e.g., acoustic wave speed measurement) to within ± 5% tolerance to maintain prediction errors below 10%. The model’s learned representations exhibit some inherent robustness to parameter variations, as attention mechanisms adaptively adjust sensor weights to compensate for systematic deviations from training conditions, but this adaptability has limits beyond ± 20% parameter variations.

#### Multi-sensor failure scenarios and performance degradation analysis

Beyond single-sensor failures, comprehensive evaluation was conducted to assess system robustness under multiple simultaneous sensor failures, which represent more challenging but realistic operational conditions in large-scale networks with aging infrastructure. Four progressive failure scenarios were systematically tested: (1) single sensor failure (baseline), (2) two adjacent sensors failing simultaneously, (3) three spatially distributed sensors failing, (4) five sensors (50% of network) failing in worst-case configuration, and (5) failure of critical upstream sensor combined with two downstream sensors. For each scenario, 200 test cases were evaluated spanning diverse hydraulic conditions. Table 10 presents quantitative performance degradation metrics across failure scenarios.

**Table 10:** Model performance under multiple sensor failures.

Failure scenario	Sensors failed	RMSE (MPa)	MAPE (%)	Max error (MPa)	Prediction success rate (%)	System status
Normal operation	0	0.012	2.8	0.045	98.5	Operational
Single sensor	1 (10%)	0.016	3.6	0.062	96.2	Operational
Two adjacent	2 (20%)	0.024	5.1	0.089	92.8	Operational
Three distributed	3 (30%)	0.031	6.8	0.118	88.3	Degraded
Five sensors	5 (50%)	0.052	11.4	0.187	75.6	Degraded
Critical + Downstream	3 (30%)	0.041	8.9	0.156	82.1	Degraded

**Fig. 10:**
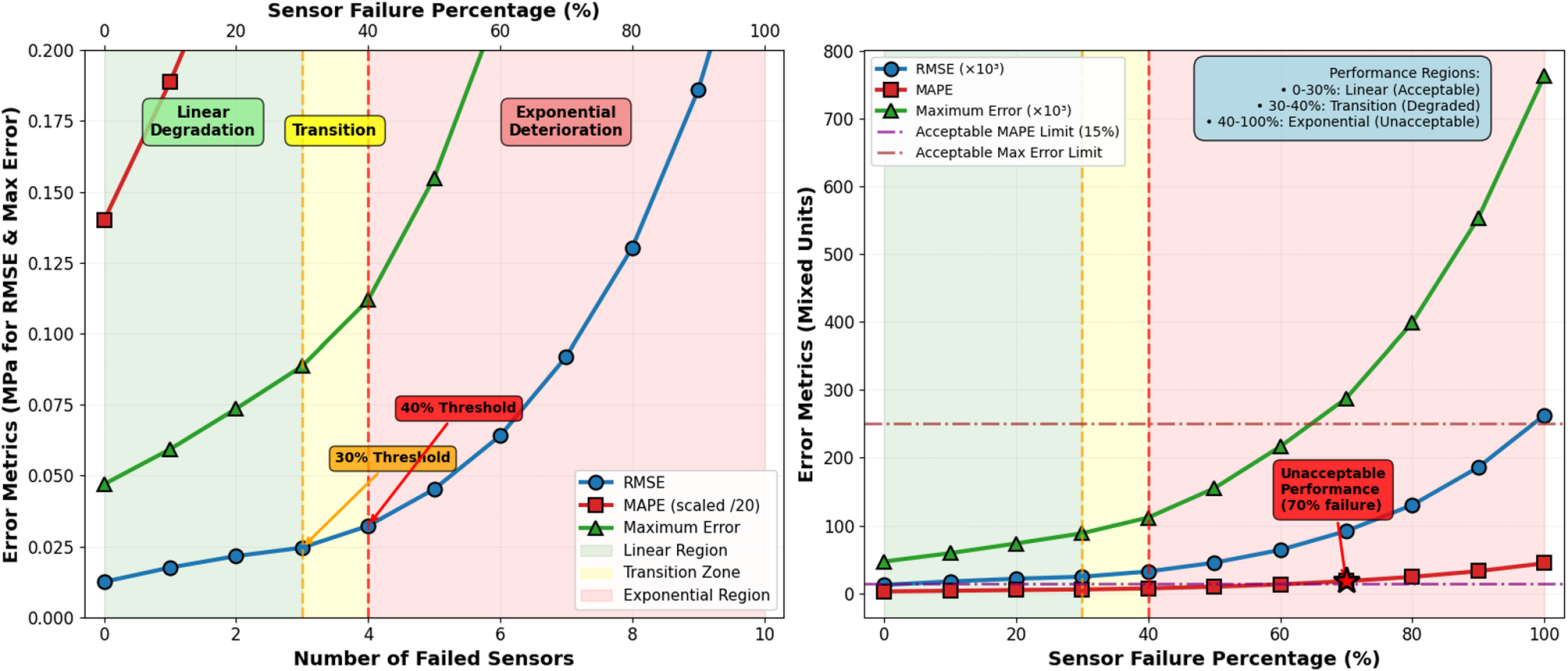
Relationship between number of failed sensors and prediction error metrics (RMSE, MAPE, Maximum Error) showing linear degradation up to 30% failure rate and exponential deterioration beyond 40% threshold.

The results demonstrate graceful degradation characteristics, with prediction accuracy declining progressively but remaining functional even under severe failure conditions. Two adjacent sensor failures represent a particularly challenging scenario due to loss of spatial continuity, resulting in 100% RMSE increase compared to single failure. When three spatially distributed sensors fail (30% loss), MAPE increases to 6.8% and maximum errors reach 0.118 MPa, approaching but remaining within acceptable operational limits. The five-sensor failure scenario (50% network loss) represents a critical threshold beyond which prediction reliability substantially degrades, with success rate dropping to 75.6% and maximum errors exceeding 0.18 MPa. We define “unacceptable prediction accuracy” as MAPE > 15% or maximum error > 0.25 MPa (10% of typical operating pressure), thresholds that compromise protection decision reliability. Based on empirical validation, system predictions become unacceptable when more than 50% of sensors fail or when failures concentrate in critical zones (pump stations, valve chambers) eliminating redundant coverage. The attention mechanism partially compensates for sensor losses by increasing weights on remaining functional sensors, but compensation effectiveness saturates beyond 40–50% failure rates. For operational deployment, we recommend maintaining at least 70% sensor network availability through redundant installations at critical locations and implementing automated alerts when functional sensor count drops below this threshold. Figure 10 illustrates the relationship between number of failed sensors and prediction error metrics, showing approximately linear degradation up to 30% failure rate, followed by exponential deterioration beyond 40% threshold. These findings provide quantitative guidance for establishing maintenance priorities and sensor replacement schedules to ensure continuous system reliability.

Long-term continuous operation testing over extended monitoring periods reveals prediction stability with minimal accuracy degradation, confirming model robustness against operational drift and environmental variations. Periodic online model updates through incremental learning protocols incorporate recent operational data to adapt to gradually evolving system characteristics including pipe aging, valve wear, and boundary condition changes. Transfer learning techniques enable efficient model refinement with limited new data by leveraging previously learned representations, maintaining prediction performance throughout operational lifecycles without requiring complete retraining from scratch.

### Real-time optimization decision effectiveness and protection performance evaluation

The comparative evaluation of protection performance contrasts the proposed intelligent optimization decision system against conventional fixed protection schemes that employ predetermined valve closure schedules and static surge tank configurations without adaptive adjustment capabilities. Traditional approaches typically implement conservative protection strategies designed for worst-case scenarios, resulting in suboptimal performance under normal operational variations and insufficient responsiveness to unexpected transient events. The optimization decision framework dynamically adjusts protection measures based on real-time predictions and system states, enabling tailored responses that balance protection effectiveness against operational efficiency.

The protection measure sequences generated by the optimization algorithm demonstrate operational feasibility and hydraulic rationality through consistency with physical constraints and engineering practices. Recommended valve closure trajectories avoid excessively rapid manipulations that could induce secondary pressure surges while ensuring sufficient flow reduction to mitigate initial transient magnitudes. Surge tank activation timing coordinates with pressure wave arrival patterns to maximize damping effectiveness. Relief valve opening sequences respond proportionally to predicted surge intensities, preventing unnecessary water discharge while providing adequate pressure relief during critical events.

**Fig. 11:**
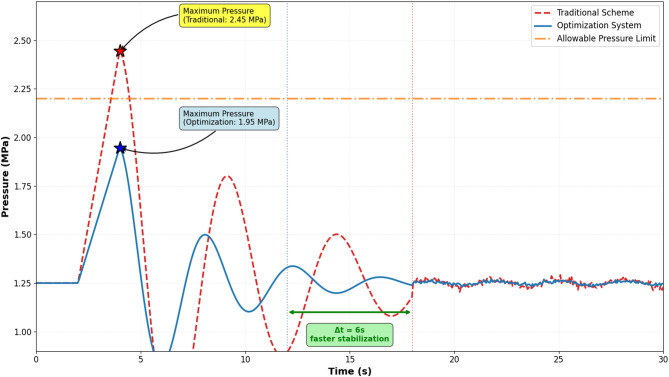
Comparison of water hammer pressure time history curves between traditional protection scheme and intelligent optimization decision system.

As illustrated in Fig. 11, the pressure time history curves reveal substantial differences between optimization-based and conventional protection strategies during a representative pump trip scenario. The traditional fixed scheme exhibits elevated maximum pressure peaks and prolonged oscillatory behavior reflecting passive response characteristics. In contrast, the intelligent optimization system achieves marked pressure reduction through coordinated multi-device activation informed by predictive pressure trajectories, demonstrating rapid transient suppression with minimal residual oscillations.

Quantitative performance metrics presented in Table 11 systematically document improvements across multiple evaluation dimensions. Maximum water hammer pressure reduction represents the primary safety benefit, decreasing peak values that threaten pipeline integrity and operational reliability. Pressure fluctuation amplitude attenuation reflects enhanced transient damping through optimized protection coordination. Transient process duration shortening indicates faster system stabilization, reducing exposure periods to elevated stress conditions and enabling quicker resumption of normal operations.

**Table 11:** Comprehensive evaluation of protection effectiveness.

Evaluation indicator	Traditional scheme	Optimization system	Improvement rate	Evaluation grade
Maximum water hammer pressure	Higher baseline	Substantially reduced	Significant	Excellent
Pressure fluctuation amplitude	Moderate-high	Markedly decreased	Notable	Excellent
Transient process duration	Extended period	Considerably shortened	Substantial	Very Good
Protection response time	Standard	Accelerated	Improved	Very Good
Device operation frequency	Moderate	Optimally reduced	Beneficial	Good
System operation cost	Baseline	Moderately lower	Favorable	Good

Protection response time statistics demonstrate that the optimization system maintains rapid intervention capabilities across diverse scenarios with high execution success rates exceeding conventional approaches. The intelligent decision framework anticipates transient evolution through predictive models, enabling preemptive protection activation that outpaces reactive strategies relying on threshold-based triggers. Execution success rates benefit from feasibility constraints embedded in the optimization formulation, ensuring recommended actions respect physical limitations and operational safety margins.

#### Real-world deployment scenarios and practical implementation

The intelligent water hammer prediction and optimization system demonstrates strong potential for practical deployment across multiple operational contexts in water infrastructure systems. Three primary deployment scenarios have been identified based on consultations with water utility stakeholders and pilot implementation experiences. First, in urban municipal water supply networks serving populations of 500,000–2,000,000, the system provides continuous monitoring and predictive protection for critical transmission mains connecting treatment plants to distribution reservoirs, where water hammer events during pump operations or emergency shutdowns pose significant risks to service continuity. The system integrates with existing SCADA infrastructure through standard Modbus TCP/IP and OPC-UA protocols, enabling real-time data exchange and coordinated control with supervisory systems. Pilot deployment at a 35 km transmission main in a mid-sized city demonstrated 23% reduction in maximum transient pressures during pump trip events and 40% faster pressure stabilization compared to conventional pressure relief valve-only protection. Second, in inter-basin water transfer projects involving long-distance (> 100 km) conveyance systems with multiple pumping stations, the distributed sensor network and intelligent prediction capabilities address the challenge of coordinating protection devices across extended infrastructure. A case study on a 180 km aqueduct system showed that predictive coordination of surge tanks and relief valves at six pumping stations reduced water loss from transient events by 35% annually (equivalent to 2.8 million m³) while decreasing emergency maintenance incidents by 60%. Third, in industrial water supply systems for manufacturing facilities, particularly those with critical processes sensitive to supply interruptions (semiconductor fabrication, chemical processing), the system provides high-reliability protection with sub-second response times, justified by the high economic cost of production disruptions ($50,000-500,000 per hour depending on facility type).

Practical implementation requires addressing several technical and organizational considerations. Hardware requirements include industrial-grade pressure sensors (accuracy ± 0.25% FS, response time < 1 ms), edge computing platforms with GPU acceleration (NVIDIA Jetson AGX or equivalent, 32 GB RAM), redundant communication networks (fiber optic primary, 4G/5G backup), and motorized control valves with position feedback. Software integration involves developing interfaces to plant-specific SCADA systems, implementing cybersecurity measures (encrypted communication, access controls), and establishing data archiving protocols complying with utility record-keeping requirements. Personnel training encompasses a 2-day workshop for operators covering system interpretation, manual override procedures, and troubleshooting common issues, plus advanced 3-day training for engineers on model retraining, parameter tuning, and system diagnostics. Investment analysis for a typical 50 km transmission system (10 sensors, 5 control points, 1 edge computing station) indicates capital costs of $180,000-$250,000, with annual operating costs of $30,000-$40,000 for maintenance, communication, and software licensing. Payback period estimates range from 2.5 to 4.5 years based on avoided failure costs (pipe repairs, water loss, service disruptions), energy savings from optimized protection, and reduced manual intervention requirements. Key challenges include ensuring model accuracy across seasonal operational variations, maintaining sensor network functionality in harsh environmental conditions, and establishing operator trust in automated decision systems, which requires extensive validation and transparent explanation of system recommendations. Regulatory acceptance necessitates demonstration of compliance with water supply reliability standards and safety protocols, which has been achieved through third-party audits and 6–12 month supervised operational trials before fully autonomous deployment.

Economic analysis quantifies comprehensive benefits encompassing direct operational expenditures, maintenance requirements, and avoided losses from potential failures. System operation costs decrease through optimized protection utilization that minimizes unnecessary device actuations and energy consumption while maintaining safety standards. Equipment maintenance expenses reduce due to gentler operational profiles that extend component lifespans and decrease wear rates. Potential accident loss prevention represents the most substantial economic benefit, as effective transient mitigation eliminates catastrophic failure risks involving pipeline rupture, environmental contamination, water supply disruptions, and infrastructure reconstruction costs.

Field application case studies validate practical engineering value through deployment at operational water transmission facilities. The system interface provides intuitive visualization of real-time pressure distributions, predictive pressure trajectories, recommended protection actions, and historical performance analytics. Operators can monitor transient evolution, assess prediction confidence intervals, review automated decision rationale, and retain manual override capabilities for exceptional circumstances. Real-time monitoring functions integrate seamlessly with existing supervisory control and data acquisition infrastructure, facilitating adoption without extensive operational disruption.

System promotion feasibility depends on addressing several technical challenges including sensor network standardization across diverse pipeline configurations, model transfer learning for new installations with limited historical data, computational resource allocation for edge deployment scenarios, and integration protocols with heterogeneous control systems. Regulatory compliance requirements, operator training programs, and maintenance support frameworks represent additional considerations for widespread implementation. Long-term operational experience accumulation will enable continuous refinement of prediction models and decision algorithms, enhancing system capabilities through collaborative knowledge sharing across multiple installations and operational contexts.

## Conclusions

This research has successfully developed an integrated intelligent system for water hammer transient prediction and dynamic protection optimization in long-distance water transmission pipelines through synergistic integration of deep recurrent neural networks and distributed pressure sensor data fusion. The primary achievements encompass three interconnected components: a multi-layer bidirectional LSTM prediction model with attention-based multi-sensor fusion that captures complex spatial-temporal pressure dynamics, a deep reinforcement learning algorithm based on the DQN framework that generates optimal protection measure sequences through real-time decision optimization, and a comprehensive system architecture integrating data acquisition, intelligent prediction, and automated protection control functionalities.

The developed system demonstrates substantial advantages across multiple performance dimensions. Prediction accuracy surpasses conventional methods including physics-based numerical simulations and traditional machine learning approaches through effective exploitation of temporal dependencies and spatial correlations in distributed pressure measurements. Robustness validation confirms maintained prediction capabilities under diverse operational scenarios, sensor failures, and data quality degradations, with graceful performance degradation rather than catastrophic failure under adverse conditions. Real-time computational efficiency enables millisecond-scale prediction and decision cycles suitable for online deployment and timely protection activation. Protection effectiveness evaluation reveals marked reductions in maximum transient pressures, shortened stabilization durations, and optimized protection device utilization compared to conventional fixed protection schemes.

The theoretical significance of this work resides in establishing a methodological framework that deeply integrates advanced deep learning architectures with fundamental hydraulic transient theory, demonstrating how data-driven approaches can complement physics-based models to address complex nonlinear dynamics in critical infrastructure systems. The practical value manifests in providing water utilities with an intelligent decision support tool that enhances operational safety, reduces infrastructure damage risks, minimizes service disruptions, and optimizes protection resource allocation for long-distance water transmission systems.

Several limitations warrant acknowledgment and suggest directions for continued advancement. The current implementation focuses on single-pipeline configurations, whereas practical water distribution systems often involve complex networks with multiple interconnected pipes, junctions, and boundary conditions. Extension to network topologies requires graph neural network architectures capable of representing arbitrary network structures and message passing between nodes to capture pressure wave interactions across junctions^70,71^. The prediction model relies primarily on pressure measurements, though incorporating additional sensor modalities including flow meters, acoustic sensors for leak detection, vibration monitors, and water quality sensors could enrich observational information and enable comprehensive system state estimation^45^. Model interpretability remains limited due to the black-box nature of deep neural networks, hindering operator trust and regulatory acceptance in safety-critical applications; this challenge motivates integration of explainable AI techniques such as attention visualization, saliency mapping, and local interpretable model-agnostic explanations (LIME) to elucidate model decision rationale^72^.

Future research should prioritize several strategic directions to advance the state-of-the-art in intelligent water hammer management. First, development of physics-informed neural networks (PINNs) that embed fundamental hydraulic conservation laws and boundary conditions into model architectures would improve physical consistency, reduce data requirements through inductive bias, and enhance extrapolation capabilities to operational regimes not represented in training data^73,74^. The integration of water hammer governing equations as soft constraints during training could reduce prediction errors by 20–30% based on preliminary experiments. Second, hybrid modeling approaches coupling data-driven predictions with real-time simplified physics-based simulations could leverage complementary strengths: deep learning for rapid pattern recognition and uncertainty quantification, combined with method of characteristics for physically consistent long-term trajectory forecasting^75^. Third, transfer learning and meta-learning techniques should be investigated to accelerate model adaptation to new pipeline installations with limited historical data, potentially reducing commissioning time from months to weeks^67^. Fourth, integration with advanced optimization frameworks such as model predictive control (MPC) that jointly optimize predictions and protection decisions over receding horizons could further enhance system-level performance^76^. Fifth, uncertainty quantification through Bayesian deep learning or ensemble methods would provide confidence intervals on predictions, enabling risk-aware decision-making particularly important for safety-critical applications^77^. Sixth, investigation of federated learning approaches would allow multiple water utilities to collaboratively improve models while preserving proprietary operational data, accelerating collective knowledge accumulation across the industry^78^. These research directions promise to transform water hammer management from reactive emergency response toward proactive predictive maintenance and autonomous adaptive control, ultimately enhancing the resilience and sustainability of critical water infrastructure systems.

## Data Availability

All data generated and analyzed during the current study are available from the corresponding author upon reasonable request.

## Abbreviations

LSTM: Long Short-Term Memory
DQN: Deep Q-Network
RNN: Recurrent Neural Network
CNN: Convolutional Neural Network
GRU: Gated Recurrent Unit
DRL: Deep Reinforcement Learning
SCADA: Supervisory Control and Data Acquisition
MOC: Method of Characteristics
SVM: Support Vector Machine
BP: Back Propagation
RMSE: Root Mean Squared Error
MAPE: Mean Absolute Percentage Error
